# Enhanced Artificial Enzyme Activities on the Reconstructed Sawtoothlike Nanofacets of Pure and Pr-Doped Ceria Nanocubes

**DOI:** 10.1021/acsami.1c09992

**Published:** 2021-08-09

**Authors:** Lei Jiang, Miguel Tinoco, Susana Fernández-García, Yujiao Sun, Mariia Traviankina, Pengli Nan, Qi Xue, Huiyan Pan, Almudena Aguinaco, Juan M. González-Leal, Ginesa Blanco, Eduardo Blanco, Ana B. Hungría, Jose J. Calvino, Xiaowei Chen

**Affiliations:** † Departamento de Ciencia de los Materiales, Ingeniería Metalúrgica y Química Inorgánica, Facultad de Ciencias, Universidad de Cádiz, Campus Río San Pedro, Puerto Real, Cádiz E-11510, Spain; ‡ Heavy Oil State Laboratory and Center for Bioengineering and Biotechnology, College of Chemical Engineering, China University of Petroleum (East China), Qingdao 266580, China; § Departamento de Física de la Materia Condensada, Facultad de Ciencias, Universidad de Cádiz, Campus Río San Pedro, Puerto Real, Cádiz E-11510, Spain; ∥ Henan Key Laboratory of Industrial Microbial Resources and Fermentation Technology, College of Biological and Chemical Engineering, Nanyang Institute of Science and Technology, Nanyang 473004, China; ⊥ Instituto Universitario de Investigación en Microscopía Electrónica y Materiales (IMEYMAT), Universidad de Cádiz, Campus Río San Pedro, Puerto Real, Cádiz E-11510, Spain

**Keywords:** ceria, nanocubes, Pr-doping, oxidation treatment, artificial enzyme, nanofacets

## Abstract

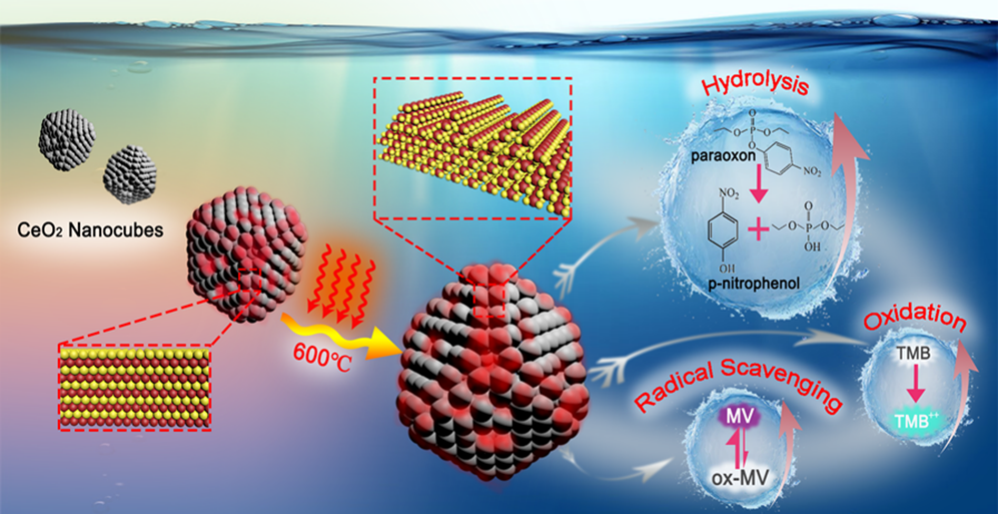

In this work, a simple one-step thermal oxidation process was established to achieve a significant surface increase in {110} and {111} nanofacets on well-defined, pure and Pr-doped, ceria nanocubes. More importantly, without changing most of the bulk properties, this treatment leads to a remarkable boost of their enzymatic activities: from the oxidant (oxidase-like) to antioxidant (hydroxyl radical scavenging) as well as the paraoxon degradation (phosphatase-like) activities. Such performance improvement might be due to the thermally generated sawtoothlike {111} nanofacets and defects, which facilitate the oxygen mobility and the formation of oxygen vacancies on the surface. Finally, possible mechanisms of nanoceria as artificial enzymes have been proposed in this manuscript. Considering the potential application of ceria as artificial enzymes, this thermal treatment may enable the future design of highly efficient nanozymes without changing the bulk composition.

## 1 Introduction

Natural enzymes play a crucial role in catalyzing almost all biochemical reactions in biological systems. Their catalytic efficiency is extraordinary in terms of selectivity, yield, and reaction rate. Because of their restraint in denaturation conditions, stability and cost, for a long time people have been intending to find a substitution of enzymes using inorganic materials, that is, artificial enzymes.^1^ Recent developments in nanomaterial synthesis and characterization have triggered many scientists to study them as artificial enzymes.^1−3^ A number of nanomaterials, such as CeO_2_,^4^ Au,^5^ Fe_3_O_4_,^6,7^ carbon nanotubes,^8^ etc., have demonstrated multienzyme-mimic functions of oxidase,^9^ peroxidase,^8^ superoxide dismutase, and phosphatase.^10,11^ Previous studies have shown that the enzymelike activities depend on the size, morphology, composition, and surface coating of nanomaterials, and consequently their activities can be tuned by controlling these parameters.^1,2,12^

Ceria is one of the most studied artificial enzymes among bare nanomaterials with intrinsic enzymatic properties.^4^ The key reason for this is often attributed to its high oxygen storage capacity and high efficiency as an “oxygen buffer”. Thus, ceria is able to release and store oxygen when it is treated alternatively under reducing and oxidizing atmospheres, by means of a facile redox Ce^3+^/Ce^4+^ cycle.^4^ Since the pioneering studies by Seal et al. and Perez et al. on nanoceria’s functions as artificial enzymes, more investigations have been focused on tuning its enzymelike kinetics and performance.^9,13−17^ Previous studies indicate that ceria with controlled morphologies presents outstanding properties, different from those of conventional ceria particles.^9,18,19^ The exposure of more active facets, which might facilitate the redox cycles, in the case of nanoceria with well-defined shapes must be at the roots of this enhancement of the enzyme-mimic activity.^20^ Doping ceria with other elements, such as other lanthanides or aliovalent cationic metal dopants, can enhance the redox properties of ceria due to an increase in the concentration of oxygen vacancies and oxygen mobility in ceria.^18,21,22^ In particular, Pr can greatly improve the oxygen storage capacity of ceria because of its redox Pr^3+^/Pr^4+^ couple.^21,23,24^

Though many reports are available on various methodologies, synthesizing enzymatic nanocrystals with controlled size and surface through a single method still remains a challenge. Yang et al. prepared CeO_2_ nanocubes (NCs) and nanorods (NRs) exposing different facets via a hydrothermal method.^25^ It was reported that at the same levels of Ce^3+^/Ce^4+^ cations and oxygen vacancies on the surface, CeO_2_ NCs with exposed {100} facets displayed higher peroxidase but lower superoxide dismutase-mimic activity than CeO_2_ nanorods with exposed {110} facets.^25^ Other researchers have found that in CO oxidation reaction ceria with defined surface planes exhibit catalytic activities in deceasing order nanorods > nanocubes > octahedral.^19,20^ Our recent work reveals that ceria nanorods without any addition of other metals as the active phase exhibit the highest catalytic activity among the ceria nanocubes, commercial ceria with high and low surface areas, and ceria nanorods for selective oxidation of glycerol in the liquid phase.^26^ Both experimental and theoretical studies have shown that oxygen vacancies are easier to form on {110} and {100} than on {111} facets.^19,20^ Despite the efforts and sometimes controversial results, it is difficult to define a facile synthesis method to control the facets of ceria nanoshapes which allows one to enhance their intrinsic enzymelike activities.

In this study, pure and Pr-doped ceria nanocubes (5, 10, and 15 mol % Pr) have been synthesized. Based on previous studies about the effects of surface reconstruction on the intrinsic catalytic activity in CO oxidation of Au supported on ceria nanocubes,^27^ a thermal treatment under oxidizing conditions was performed on both ceria nanocubes. Such treatment leads to the transformation of {110} surfaces of the nanocubes into a system of {111} nanofacets, without significantly changing their overall, cubelike morphology. This study continues to systematically explore the influence of such defects on ceria’s artificial enzyme activities, including oxidase, hydroxyl radical scavenger, and phosphatase. Knowing the importance in catalysis of crystal defects on edges and corners of nanoparticles, this study provides us an easy tool in order to control nanofacets of ceria nanocubes with a view to manipulating their enzymatic activities.

## 2 Experimental Section

### 2.1 Synthesis of Pure and Pr-Modified Ceria Nanocubes

CeO_2_ nanocubes (CeO_2_NC), together with 5, 10, and 15 mol % Pr-CeO_2_ nanocubes (namely, 5% Pr-CeO_2_NC, 10% Pr-CeO_2_NC, and 15% Pr-CeO_2_NC), were synthesized by the same hydrothermal method described in a previous paper.^21^ Ceria NCs with reconstructed nanofacets, namely, CeO_2_NC-O600, 5% Pr-CeO_2_NC-O600, 10% Pr-CeO_2_NC-O600, and 15% Pr-CeO_2_NC-O600, were prepared by oxidizing the CeO_2_NC, 5% Pr-CeO_2_NC, 10% Pr-CeO_2_NC, and 15% Pr-CeO_2_NC samples in a 60 mL min^–1^ flow of 5% O_2_/He at 600 °C for 1 h. Afterward, the samples were cooled down to room temperature under the same oxidizing mixture.

### 2.2 Characterization of Catalysts

The pure and Pr-doped ceria nanocube samples were characterized by powder X-ray diffraction (XRD), X-ray photoelectron spectroscopy (XPS), inductively coupled plasma-atomic emission spectroscopy (ICP-AES), Raman spectroscopy, and transmission electron microscopy (TEM) techniques. XRD patterns were recorded on a D8 ADVANCE diffractometer of Bruker using Cu Kα radiation. The intensity data were collected over a 2θ range of 20–70°. The average crystallite size of all the oxides was estimated by the Scherrer equation using the width of the {111} diffraction peak of ceria at 28.6°. Likewise, the lattice parameters of all the ceria nanocubes were calculated using the Bragg equation.

The actual Pr content of the doped nanocubes was analyzed using ICP-AES equipment (Iris Intrepid, Thermal Elemental). The Brunauer–Emmett–Teller (BET) surface areas of the samples were determined by N_2_ physisorption at −196 °C on a Micromeritics ASAP 2020.

XPS measurements were performed on a Kratos Axis Ultra DLD instrument to characterize the surface chemical composition and oxidation state of the samples. The spectra were collected using monochromatized Al Kα radiation (1486.6 eV), with an X-ray power of 150 W. The spectrometer was operated in the constant analyzer energy mode, with a pass energy of 20 eV. The powder samples were pressed to prepare self-supported pellets, which were stuck on a double-sided adhesive conducting polymer tape. Surface charging effects were compensated by making use of the Kratos coaxial neutralization system. The binding energy (BE) scale was calibrated with respect to the C 1s signal at 284.8 eV.

All the samples were characterized by high-resolution transmission electron microscopy (HRTEM) and scanning transmission electron microscopy-high angle annular dark field (STEM-HAADF) using a JEOL 2010-F microscope and an aberration-corrected FEI Titan^3^ Themis 60-300 microscope. HRTEM images were obtained with 0.19 nm spatial resolution at Scherzer defocus, and STEM-HAADF images were collected by using an electron probe of 0.5 nm of diameter at a camera length of 8 cm. Additionally, high spatial energy dispersive X-ray spectroscopy (XEDS) maps were acquired using the ChemiSTEM capabilities of a FEI Titan^3^ Themis 60-300 microscope. In this case, a high brightness, subangstrom (0.07 nm) diameter electron probe was combined with a highly stable stage to record these maps. Element mapping was acquired with a screen current of 200 pA and a pixel dwell time of 170 μs. This dwell time resulted in a frame acquisition time of approximately 25 s, after which the drift was corrected using cross correlation. An averaging filter was used on the images as provided in the Esprit software.

Raman spectra of the samples were recorded using a confocal dispersive Raman spectrometer (Jasco, model NRS-7200) in backscattering configuration. A 532 nm Nd–YAG laser operating at 5.6 mW power was used as an excitation source. The laser beam was focused on the samples by a 100× microscope objective, with a spot size of about 1 μm diameter.

The reducibility of ceria nanocubes was studied by temperature-programmed reduction analysis with H_2_ (H_2_-TPR). About 150 mg of the powder was oxidized under a 5% O_2_/He flow (60 mL min^–1^) at 500 °C for 1 h and cooled in the same oxidizing flow down to 150 °C. Then, the flow was switched to He in order to remove adsorbed oxygen species on the surface of the samples and cool down the sample to room temperature. Afterward, the He flow was replaced by a flow of 5% H_2_/Ar at room temperature. The H_2_-TPR experiments were carried out in a 60 mL min^–1^ flow of 5% H_2_/Ar with a heating rate of 10 °C min^–1^ from room temperature to a maximum temperature of 950 °C, keeping the sample at this temperature for 1 h. The reactor effluent gas was passed and analyzed by a Thermostar GSD301T1 mass spectrometer from Pfeiffer Vacuum. The mass/charge ratio (*m*/*z*) value used to monitor H_2_O formation is 18 during the H_2_-TPR process.

In order to study the effect of Pr modification and the oxidation treatment at 600 °C on the optical properties of ceria nanocubes and estimate their energy band gap, UV–vis specular and diffuse reflectance measurements were carried out using an Agilent Cary 5000 UV–vis-NIR double-beam spectrophotometer. The spectra in the 200–2500 nm range were registered in an integrating sphere. A reference sample Pr_6_O_11_ was also studied by this technique. The resulting diffuse reflectance spectra were transformed into apparent absorption spectra using the Kubelka–Munk function (*F*(*R*)). The direct and indirect optical band gaps of the materials were determined through the construction of Tauc plots by plotting (*F*(*R*)*hν*)^*n*^ against (*hν*), with *n* = 2 or *n* = 1/2, for direct and indirect transitions, respectively. The optical band gap was obtained by extrapolating the linear part of this plot to the energy axis.

### 2.3 Artificial Enzyme Catalytic Activities

All the chemicals used in this section were purchased from Sigma-Aldrich (U.S.). The oxidase-like activity of the samples was tested for the oxidation of 3,3′,5,5′-tetramethylbenzidine (TMB).^21^ A TMB solution (0.009 M) was prepared by diluting TMB stock (0.1 M) solution in dimethyl sulfoxide (DMSO) with acetate buffer (pH = 4.0, 0.01 M). The TMB solution was then stirred thoroughly to avoid precipitation. The nanoceria suspension (0.1 mg mL^–1^) was prepared by dispersing ceria nanocubes in Milli Q water after washing and centrifuging the sample at 12000 rpm for 10 min three times. The nanoceria suspension was sonicated for 2 h before each measurement. The UV–vis absorption spectra of TMB at 652 nm (Shimadzu UV-2450 spectrophotometer) before and after the addition of nanocubes at 40 min were obtained. Steady kinetic assays on absorbance change over time were monitored in a time course mode using a SpectraMax M2e microplate reader (Molecular Devices, U.S.), where the concentration of ceria and TMB in solution (200 μL) were the same as measured with the UV–vis spectrometer.

The hydroxyl radical scavenging ability was studied by investigating their capability to remove the hydroxyl radicals generated from the Fenton reaction as previously described.^18^ The reaction solution used for the photometric determination contained methyl violet (MV, 2.4 × 10^–5^ M), FeSO_4_ (3 × 10^–4^ M), and H_2_O_2_ (0.4 M) in Tris-HCl (0.1 M, pH = 4.4) buffer. Then, ceria NCs with final concentration 1.7 μg mL^–1^ were added to the Fenton reaction solution, the mixture was blended for 1 min at room temperature and followed by the addition of MV and UV–vis absorbance measurements at 584 nm to quantify the concentration of MV. To eliminate the possible influence of ceria’s catalase-like activity, in a comparative experiment 21.1 μg mL^–1^ catalase (Aladdin, China) was added together with 1.7 μg mL^–1^ ceria NCs in a similar manner as above.

The phosphatase-like activity of nanoceria was quantified by measuring their efficiency of hydrolytically cleaving the organophosphorus compound paraoxon into *p*-nitrophenol.^10^ This was regarded as mimicking the natural enzymes like phosphotriesterase in bacteria that was able to break down organophosphorus-based nerve agents.^28^ The paraoxon was first dissolved in acetone as stock solution (2 M). CeO_2_NCs with a final concentration of 5 mg mL^–1^ (29 mM) were added to paraoxon (0.05 M) in *N*-methylmorpholine (NMM) solution (0.45 M). The mixture was thoroughly stirred and the UV–vis spectra and absorbance change over time was measured using a UV–vis spectrometer. The specific absorbance peak of *p*-nitrophenol at 401 nm was monitored. For comparison, CeCl_3_ (29 mM) and Ce(SO_4_)_2_ (29 mM) solutions were also tested for the hydrolysis effect.

## 3 Results

### 3.1 Surface and Structure Characterization

**Figure 1:**
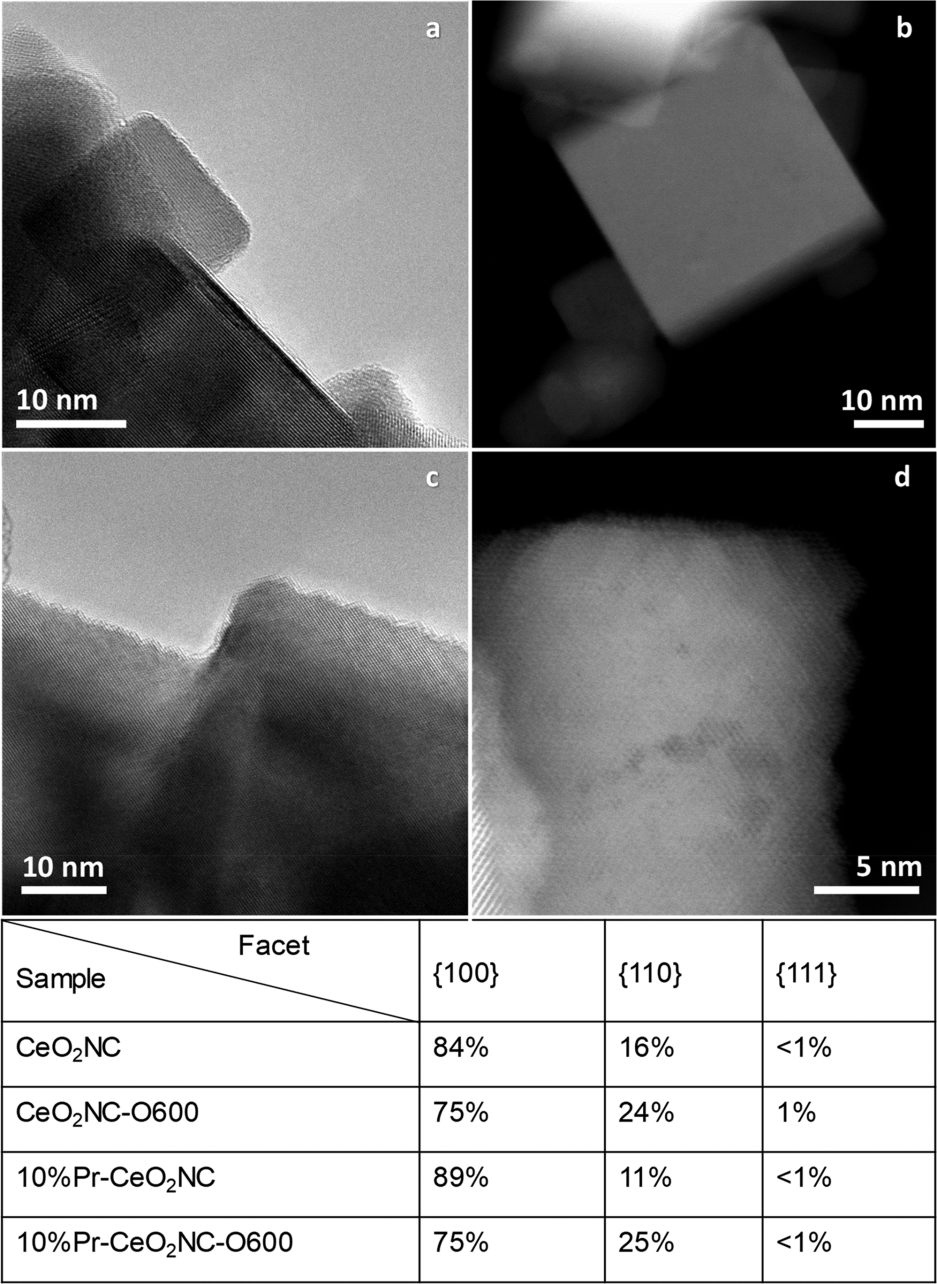
HRTEM images of (a) CeO_2_NC and (c) CeO_2_NC-O600, STEM-HAADF images of (b) 10% Pr-CeO_2_NC and (d) 10% Pr-CeO_2_NC-O600 samples. The inset table shows the percentage of the {100}, {110}, and {111} facets located at the surfaces, edges, and vertexes of the nanocubes, respectively, measured by TEM and STEM-HAADF techniques.

**Figure 2:**
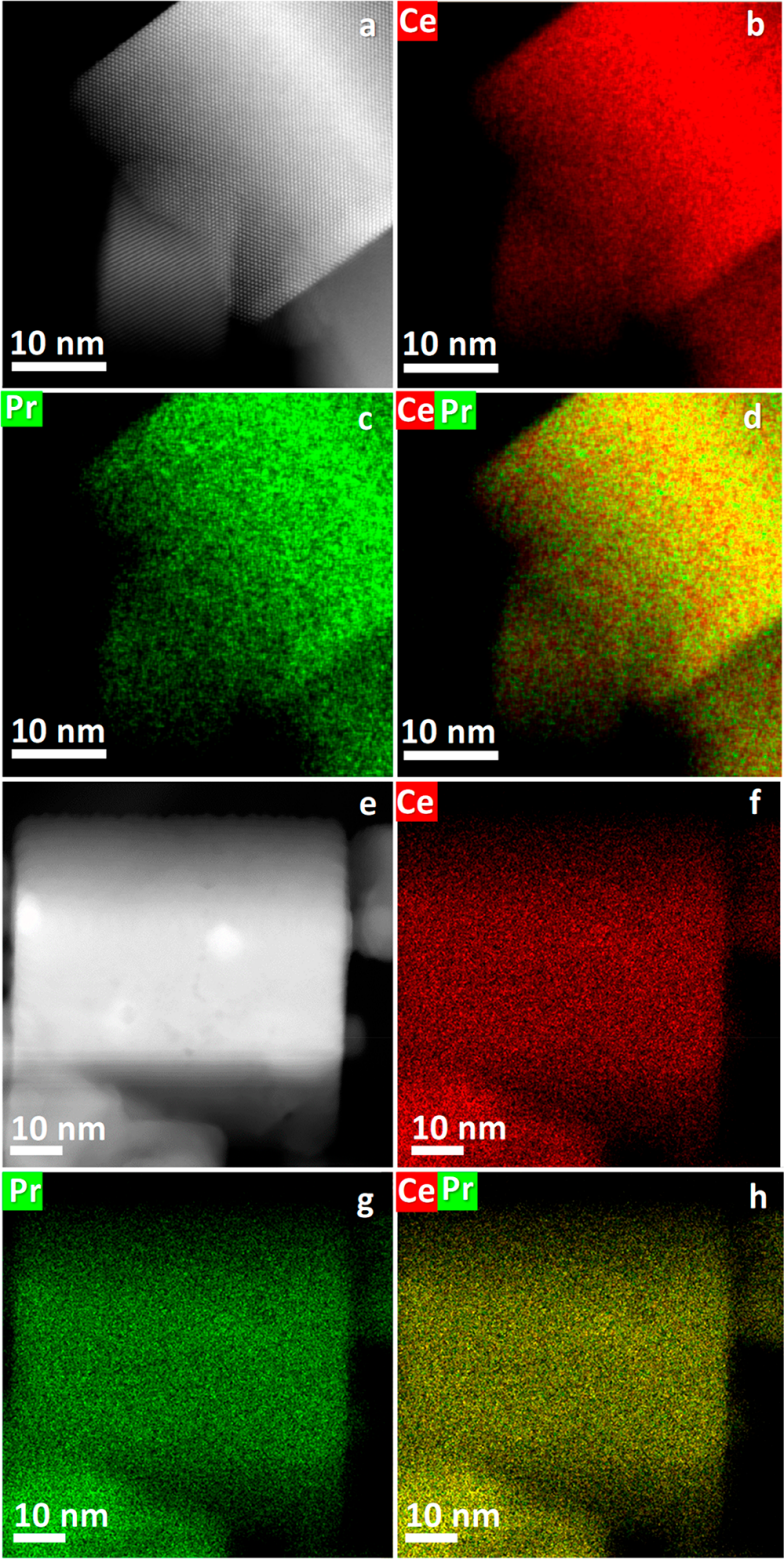
STEM-HAADF images and XEDS element maps of Ce, Pr and Ce–Pr of (a–d) 10% Pr-CeO_2_NC and (e–h) 10% Pr-CeO_2_NC-O600.

The low-magnification TEM or STEM-HAADF images of all pure and Pr-doped ceria are shown in Figure S1. It can be clearly confirmed that all these samples possess a cubic morphology. In addition, the particle sizes of these pure ceria nanocube and Pr-modified ceria nanocube samples are mainly between 5 and 50 nm (Figure S2). Figure 1 presents high-resolution TEM or STEM-HAADF images of four representative samples CeO_2_NC, CeO_2_NC-O600, 10% Pr-CeO_2_NC, and 10% Pr-CeO_2_NCO-O600. Element mapping of the 10% Pr-modified ceria nanocubes before and after oxidation in Figure 2 shows that Pr and Ce distribute homogeneously in the nanocubes and that the Pr concentration is around 10 mol %.

Furthermore, it was found that after oxidation at 600 °C, the ceria nanocube surfaces are roughened into sawtoothlike structures in both pure and Pr-modified ceria nanocubes when they are looked along the {110} zone axis (Figures 1c,d and 2e). These zigzag structures, or reconstructed {111} nanofacets, appear on both oxidized CeO_2_NC-O600 and 10% Pr-CeO_2_NC-O600 samples. The contributions of the different exposed surfaces, in percentage, calculated using TEM and STEM-HAADF images, are listed in the table inset of Figure 1. A significant fraction of the {100} surfaces, 8% for CeO_2_NC and 14% for 10% Pr-CeO_2_NC, has been converted to {110} facets. Nevertheless, as observed in the TEM images, these {110} surfaces are, in fact, mainly sawtoothlike {111} nanofacets of 1–3 nm in length.^29,30^ Meanwhile, in the case of the pure ceria, the percentage of {111} facets slightly increase to 1%, corresponding to truncations at the cube corners. For both Pr-doped NCs before and after oxidation, the {111} surface maintains less than 1%. In addition, sawtoothlike nanofacets have also been observed over the oxidized 5% Pr-CeO_2_NC-O600 and 15% Pr-CeO_2_NC-O600 samples (Figure S3). Figure S4 shows the element maps of Ce and Pr of 5% Pr-CeO_2_NC and 15% Pr-CeO_2_NC before and after oxidation at 600 °C, indicating that Pr distributes homogeneously in ceria nanocubes. The Pr concentrations in these samples are similar to their nominal values.

The structural, textural, and surface properties of pure and Pr-doped ceria nanocubes before and after oxidation at 600 °C were also characterized by XRD, N_2_ physisorption, and XPS. Figure S5 in the Supporting Information shows XRD patterns of the samples before and after oxidation at 600 °C, confirming the expected fluorite structure of ceria. The incorporation of Pr in ceria does not cause any change of lattice parameter, which is maintained at 5.4 Å for the samples (Table 1 and Table S1). This can be attributed to the almost identical radius of Pr^4+^ (96 pm) and Ce^4+^ (97 pm) cations.

**Table 1:** Physicochemical Properties of the Samples

			composition by ICP (mol %)		composition by XPS (mol %)					
sample	BET surface areas (m^2^ g^–1^)	calculated surface area (m^2^ g^–1^)^a^	Ce	Pr	Ce	Pr	Ce^3+^ in total amount of Ce (%)^b^	average particle size (nm)^a^	τ Scherrer (nm)^c^	lattice parameter (Å)^c^
CeO_2_NC	38	33					9	22	21	5.4
CeO_2_NC-O600	18	19					7	31	37	5.4
10% Pr-CeO_2_NC	25	23	90.3	9.7	90.6	9.4	2	27	29	5.4
10% Pr-CeO_2_NC-O600	20	18			89.2	10.8	0	37	41	5.4

a Calculated from the particle size distribution, including around 100 nanoparticles, obtained by TEM.

b Calculated using XPS data.

c Calculated using XRD data.

The average particle size was calculated from TEM and XRD data, as shown in Table 1. In the case of CeO_2_NC, it increases from 22 to 31 nm after oxidation at 600 °C and from 27 to 37 nm in 10% Pr-CeO_2_NC, according to TEM results. The average particle size of the nanocubes calculated by XRD data is quite close to those obtained by TEM data. The increase of particle size after oxidation at 600 °C is possibly caused by the partial agglomeration induced by the thermal treatment. This hypothesis is confirmed by N_2_ physisorption results. In fact, BET specific surface areas of the samples decreased from 38 to 18 m^2^ g^–1^ for CeO_2_NC and from 25 to 20 m^2^ g^–1^ for 10% Pr-CeO_2_NC. The surface areas calculated using TEM data for a nontruncated cubic model, which takes into account the cube length distribution in both samples, provide estimates of the surface area very similar to N_2_ physisorption results and show the same trend with respect to the effect of the oxidizing treatment. The BET surface areas of 5% Pr-CeO_2_NC and 15% Pr-CeO_2_NC samples (Table S1) are 26 and 23 m^2^ g^–1^, which are very similar to that of Pr-doped ceria nanocubes with 10% Pr.

XPS spectra of the four representative ceria samples are displayed in Figure S6 of the Supporting Information. Ce 3d peak shapes of the four samples are quite similar, and only very small amount of Ce^3+^ were present on the surface of nanocubes, in agreement with previous reports on little Ce^3+^ percentage in nanoceria.^18,21,31^ This was especially true in the case of 10% Pr-CeO_2_NC-O600 sample, in which no Ce^3+^ is detected. Concerning Pr oxidation state, 10% Pr-CeO_2_NC sample showed 100% of Pr^3+^, whereas after oxidizing at 600 °C, a small amount of Pr^4+^ could be detected on the surface. Table 1 lists the bulk and surface composition of the Pr-modified samples analyzed by ICP and XPS. In agreement with the XEDS analysis before and after thermal treatment, the concentration of Pr in the bulk and surface is around 10 mol %. All these data suggest that Pr distributes homogeneously in ceria nanocubes, both before and after oxidation at 600 °C. Table S1 also shows the ICP and XPS results of 5% and 15% Pr-doped ceria nanocubes. Their Pr concentrations according to ICP results are very close to the nominal values. However, the Pr concentrations on the surface of these two samples are 11.9 and 25.1%, which are higher than theoretical values.

### 3.2 Optical Properties

**Figure 3:**
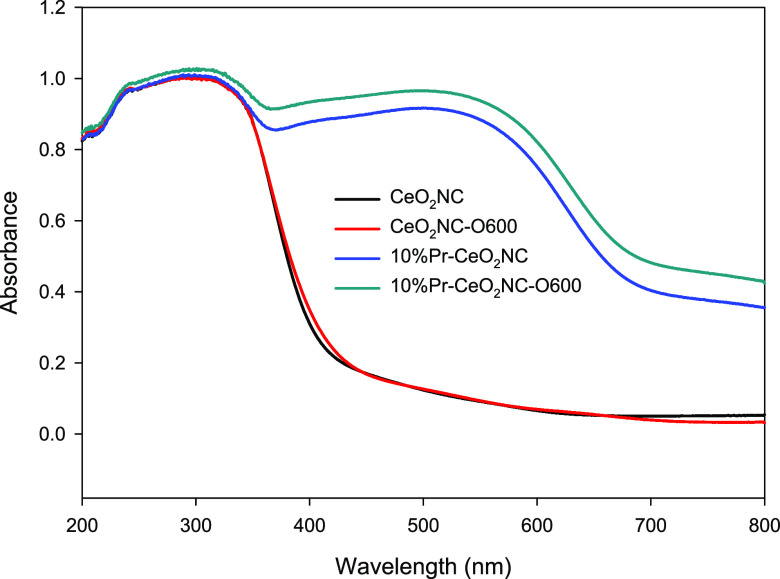
UV–vis absorption spectra of the ceria nanocube samples.

UV–vis specular and diffuse reflectance measurements were carried out, and the band gaps of four representative samples could therefore be estimated. A strong absorption around 320 nm was observed on all the samples in Figure 3, which could be assigned to the charge transfer transition from O 2p to Ce 4f.^32^ In Pr-doped samples, a broad absorption peak appears between 400 and 600 nm which is absent in pure CeO_2_ nanocubes. The appearance of this peak might be related to the interfacial polaron effect emerging from electron–phonon interaction^33^ and increased oxygen vacancies when some Ce^4+^ ions in the lattice are replaced by Pr^3+^ ions.^34^ The latter can be represented by Kroger–Vink notation (eq 1) where Ce_Ce_^*x*^ is Ce^4+^ in the lattice, VO^••^ is the oxygen vacancy, and O_O_ is the O^2–^ ion on the respective lattice site.
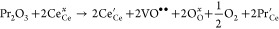
1From the UV diffuse reflectance results, the indirect and direct band gap energies of the studied materials were determined by using the Kubelka–Munk function as shown in Figure S7 in the Supporting Information. In Table 2, both indirect and direct band gap values are obtained following the procedure described in the Experimental Section. As it can be seen, the fundamental band gap of the studied materials can be assigned in all cases to the indirect one, since it is smaller than the direct one.^35^

**Table 2:** Indirect and Direct Band Gaps of All the Samples and Raman *I*_D_/*I*_F2g_ Ratios of Pr-Doped Ceria Nanocubes

catalysts	indirect *E*_g_ (eV)	direct *E*_g_ (eV)	Raman *I*_D_/*I*_F2g_
CeO_2_NC	3.17 ± 0.05	3.50 ± 0.05	
CeO_2_NC-O600	3.19 ± 0.06	3.49 ± 0.05	
10% Pr-CeO_2_NC	2.95 ± 0.03	3.53 ± 0.04	0.25
10% Pr-CeO_2_NC-O600	2.86 ± 0.06	3.51 ± 0.05	0.38

As shown in Table 2, the direct band gap energies of all the samples are around 3.50 eV, within the 3.2–3.6 eV range^36−38^ that was previously reported for ceria nanocrystals in the literature. This value is notably higher than that of bulk CeO_2_ nanocrystals (3.15 eV), probably mainly because of the quantum confinement effect.^36^ The value of direct band gap energies does not seem to be affected neither by the oxidation treatment at 600 °C nor by the addition of Pr to the ceria nanocubes.

The indirect band gap energies are systematically lower than direct ones in an amount depending on the sample. The indirect band gap of pure ceria nanocubes with or without oxidation at 600 °C is lower than the direct band gap by around 0.3 eV. The indirect band gap values decrease from 3.2 to 2.9 eV, when Pr is doped in the ceria nanocubes, which is in accordance with the values of the indirect band gap (2.9–3.3 eV) in the literature.^36−38^ Furthermore, the indirect band gap energy is even lower when the 10% Pr-CeO_2_NC sample is oxidized at 600 °C. It has been reported that the indirect band gap may be due to the band shifting from crystal structure truncation and atomic structure distortion on the nanoparticle surface.^39,40^ For the ceria nanocubes in this work, if the indirect band gap results are mainly from the surface distortion and thus higher lattice vibration, the smaller indirect band gap may suggest that the Pr doping and the subsequent thermal treatment can effectively help the indirect electron transitions from the valence band (VB) to the conduction band (CB).

**Figure 4:**
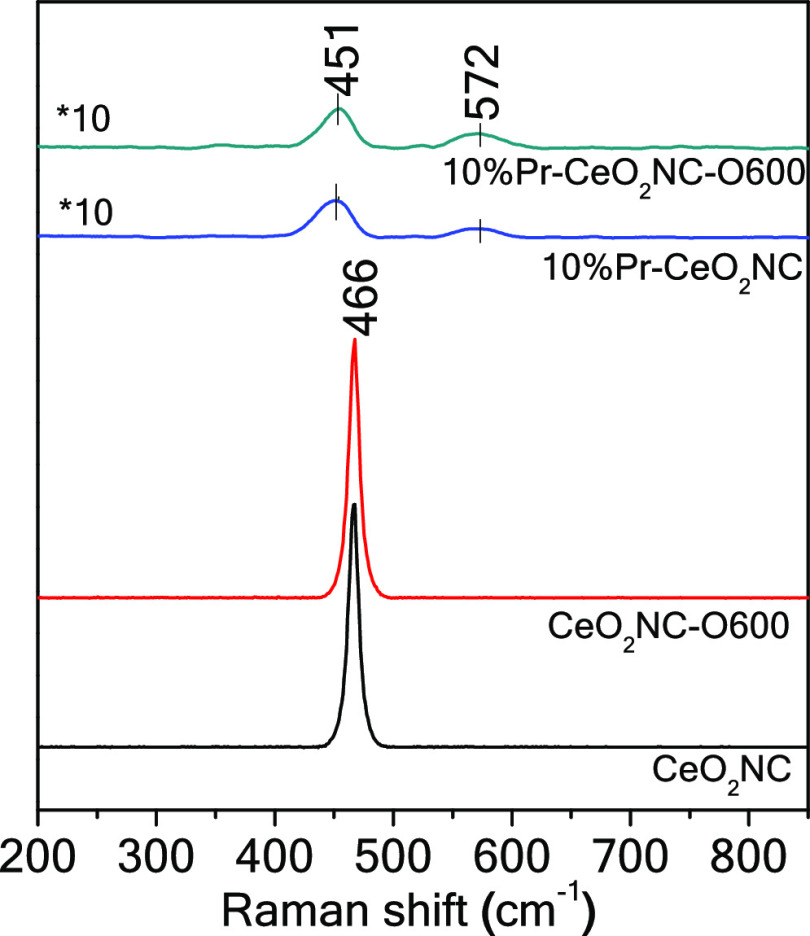
Raman spectra of the ceria nanocube samples.

Raman spectroscopy is frequently used to directly characterize the oxygen vacancies and defects in ceria. Figure 4 depicts Raman spectra of four representative ceria nanocube samples. Raman band observed at 466 cm^–1^ in both CeO_2_NC and CeO_2_NC-O600 samples is reported to be attributed to the F_2*g*_ symmetrical stretching vibration mode of CeO_2_ in the symmetrical breathing mode of the Ce–O8 vibration unit.^34^

When the ceria nanocubes are modified with Pr before and after oxidation pretreatment, a clear shift of the F_2*g*_ band to lower frequencies of 451 cm^–1^ is observed. This shift may be attributed to two consequences of Pr modification. The first one is the increased oxygen vacancies (VO^••^) when every two Pr^3+^ cations replace a Ce^4+^ cation. The second one is the dilation or contraction of the lattice.^41^ The changes in the Ce–O and Pr–O bond length and atomic geometry induce the shifting of the F_2*g*_ band. Furthermore, full width half-maximum (fwhm) values have been determined to be 10 and 11 cm^–1^ for CeO_2_NC and CeO_2_NC-O600 samples, while fwhm values for Pr-doped samples have been found to be 35 and 30 cm^–1^ for samples without and with oxidation pretreatment, respectively. The F_2*g*_ band becomes wider than that of pure CeO_2_NC, probably due to the generated VO^••^ and lattice strain.

Other than the shift of the F_2*g*_ band, a pronounced D band invariably appears at 572 cm^–1^ for both Pr-modified CeO_2_NCs. This D band can be assigned to oxygen vacancies and defect sites.^42−44^ If the intensity of the F_2*g*_ band (*I*_F_2*g*__) is used as an internal standard, the relative intensity ratio of *I*_D_/*I*_F_2*g*__ can be a good indicator for the amount of bulk oxygen vacancies, where *I*_D_ is the intensity of the D band.^41,43,45^ As shown in Table 2, the *I*_D_/*I*_F_2*g*__ value of 10% Pr-CeO_2_NC-O600 is higher than that of the unoxidized sample, suggesting that the 600 °C treatment may have caused an increase of VO^••^ and defect sites in the Pr-doped nanocubes. It is also worth noting that Raman spectra of the measured samples may vary depending on the excitation wavelength and the sample absorbance capability.^46^ For CeO_2_NC and CeO_2_NC-O600, they have no absorbance at 532 nm, which is the excitation wavelength for Raman spectra from UV–vis spectra in Figure 3. Therefore, their Raman spectra largely represent the signal from the bulk structure. On the contrary, 10% Pr-CeO_2_NC and 10% Pr-CeO_2_NC-O600 can strongly absorb the 532 nm excitation light, which means that their Raman signals are mainly from the nanocube surface. Therefore, the observed values of the intensity ratios clearly confirm an increase of the concentration of surface defects after the thermal treatment, in good agreement with the TEM/STEM results.

### 3.3 Redox Properties

**Figure 5:**
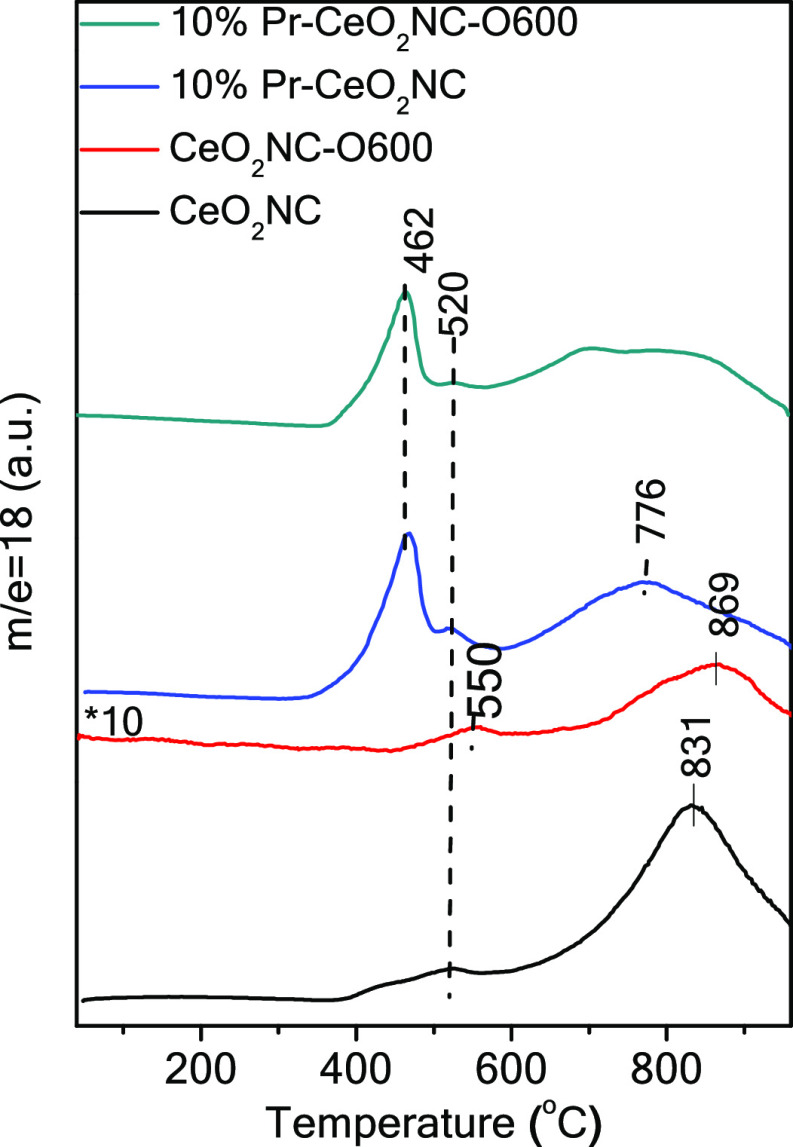
TPR profiles of the different ceria nanocube samples. The *m*/*z* = 18 signal in H_2_-TPR corresponds to H_2_O evolution.

The redox properties of the four representative samples were characterized using H_2_-TPR experiments. Figure 5 depicts the H_2_O formation during H_2_-TPR, in which oxygen is taken out from the surface or the bulk of the samples. Two reduction peaks at 520 and 831 °C, which can be attributed to the reduction of the surface and the bulk of ceria, are observed in the CeO_2_NC sample. The oxidation at 600 °C of the pure ceria nanocube sample leads to a slight shift of both peaks to higher reduction temperatures, 550 and 869 °C. However, three reduction events, at 462, 520, and 776 °C, are observed in the 10% Pr-CeO_2_NC sample. As previously reported, the Pr incorporation in NCs increases the reducibility, shifting the reduction peaks to lower temperatures.^21^ After oxidation at 600 °C, the TPR profile of the 10% Pr-CeO_2_NC-O600 sample is quite similar to that of the original sample, with a broader reduction peak at lower temperatures. This shoulder shifting to a lower temperature suggests that the treated 10% Pr-CeO_2_NC-O600 is to some extent easier to be reduced than the untreated one.

### 3.4 Biomimetic Enzyme Activities

The biomimetic enzyme activities of nanoceria have been studied previously with wide application in biomedicine and environmental protection.^1,4^ Depending on the aqueous solution conditions, nanoceria exhibits versatile performances, such as mimicking oxidase, superoxide dismutase, peroxidase, hydroxyl radical scavenger, phosphatase, etc.^1,47^ In order to investigate the effects of Pr modification and thermal treatment on CeO_2_NCs, three distinctive and characteristic capabilities, mimicking oxidase, hydroxyl radical scavenger, and phosphatase, which represent the oxidizing, reducing, and hydrolytic properties, respectively, have been evaluated in this work.

**Figure 6:**
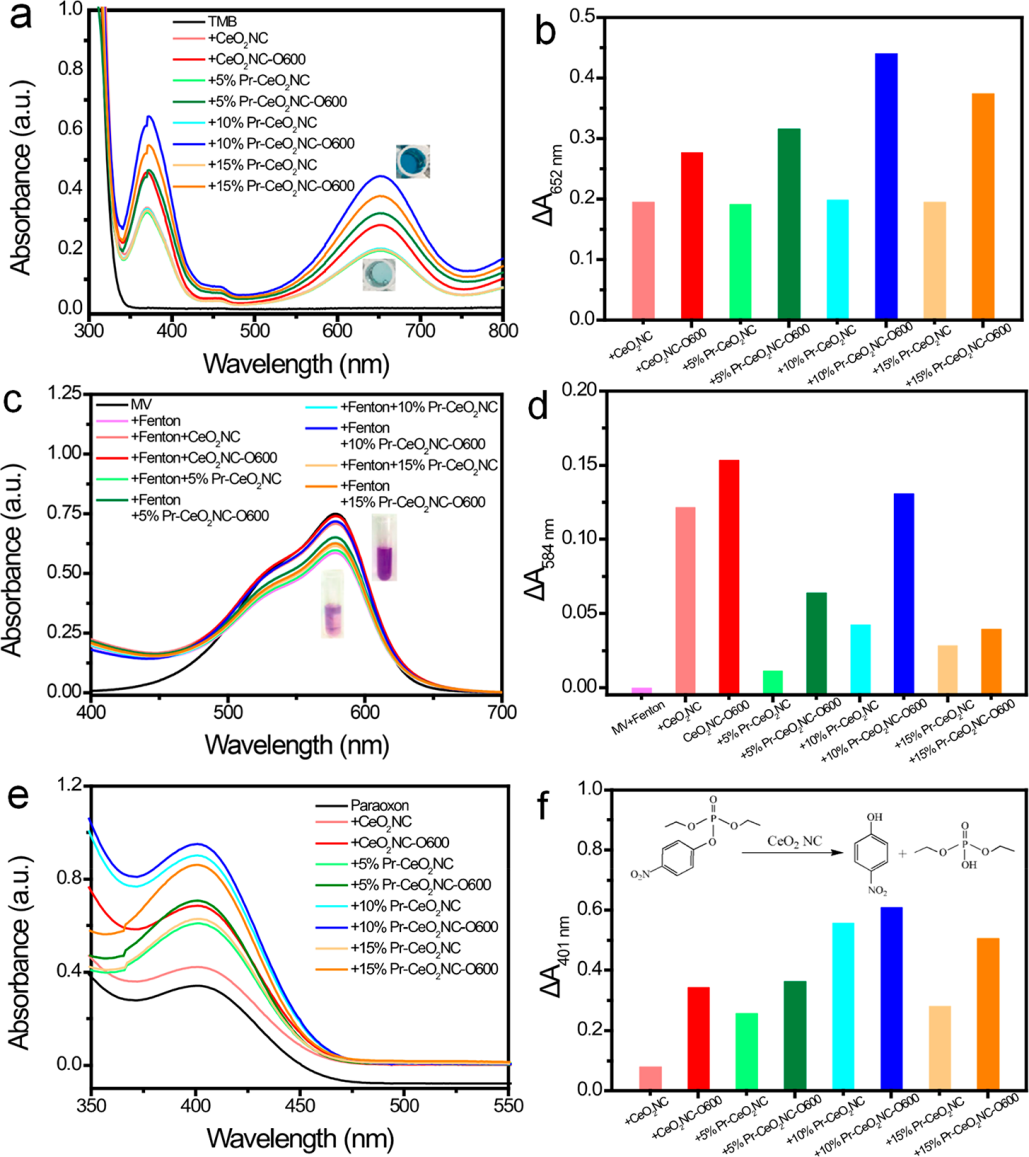
Activities of CeO_2_NC, CeO_2_NC-O600, 5% Pr-CeO_2_NC, 5% Pr-CeO_2_NC-O600, 10% Pr-CeO_2_NC, 10% Pr-CeO_2_NC-O600, 15% Pr-CeO_2_NC, and 15% Pr-CeO_2_NC-O600 as enzyme-mimics of oxidase, hydroxyl radical scavenger, and phosphatase. (a) UV–vis spectra of TMB (0.009 M) after mixing with ceria (0.1 mg mL^–1^) samples in pH 4.0 buffer. Inset photos show the color change when TMB is oxidized. (b) Comparison of the performance of the eight ceria samples in TMB oxidation; (c) UV–vis spectra of MV (2.4 × 10^–5^ M) after mixing with Fenton reagent (FeSO_4_ and H_2_O_2_), with and without the presence of different ceria (1.7 μg mL^–1^) samples. Inset photos show the color recovery as the Fenton-generated hydroxyl radicals are removed. (d) Comparison of the performance of the eight ceria samples in eliminating hydroxyl radicals; (e) UV–vis spectra of paraoxon (0.05 M) in the presence of 5 mg mL^–1^ (29 mM) ceria samples. The absorbance peak at 401 nm is from the hydrolysis product *p*-nitrophenol. (f) Comparison of the performance of the eight ceria samples in hydrolyzing paraoxon, catalytic reaction shown in the inset.

The oxidase-like activity was examined by the TMB oxidation experiment.^21^Figure 6a shows that upon the addition of ceria nanocubes, the colorless TMB solution quickly turns blue, which is attributed to the generation of blue-colored oxidized TMB with absorbance at 652 nm. Figure 6b compares the 652 nm absorbance change for the eight types of nanocubes. On one hand, Pr-modification slightly improves the oxidizing efficiency. On the other hand, for pure and Pr-doped ceria NCs, independent of the Pr concentrations in ceria nanocubes, after oxidation at 600 °C their performance is largely enhanced, with the oxidization yield increasing more than 50%. Considering the activity per unit surface area of four representative samples, the differences are even more remarkable, as shown in Figure S8a.

The antioxidant activity of the ceria samples was also investigated by measuring their capability to eliminate hydroxyl radicals as described previously.^18^Figure 6c uses absorbance of MV (2.4 × 10^–5^ M) with a purple color at 584 nm as an indicator which can be oxidized by the hydroxyl radicals generated by Fenton reagents, Fe^2+^ and H_2_O_2_, *in vitro*. After addition of the Fenton reagent (3 × 10^–4^ M FeSO_4_ and 0.4 M H_2_O_2_), MV was oxidized to colorless ox-MV, a process which can clearly be inhibited by adding 10% Pr-CeO_2_NC (1.7 μg mL^–1^). The absorbance difference Δ*A* of MV+Fenton solution without and with adding NCs is linearly related to the amount of eliminated radicals, which suggests a direct relationship between the scavenging activity and reducibility of nanoceria. The Δ*A* in the presence of the eight NC samples in Figure 6d shows that the oxidized NCs, again, are more active in removing radicals than the untreated ones. Similar results are obtained by comparing Δ*A* normalized by the surface area of nanocubes (Figure S8b). This is in accordance to the enhanced oxidase activity in Figure 6a,b. It is worth mentioning that the scavenging ability of Pr-doped CeO_2_NC is not as good as that of CeO_2_NC, and the same tendency is observed for the oxidized samples. This means that although Pr-doping can enhance the oxidizing capacity of CeO_2_NC, in the meantime, it weakens the reducing capacity of ceria which is contrary to our previous studies on La-doped CeO_2_ nanocubes.^18^ Furthermore, considering the possible influence of catalase-like activity of nanoceria that was previously reported,^48^ a comparative MV experiment was carried out in which 21.1 μg mL^–1^ (∼4.2 unit) natural catalase (CAT) was added together with CeO_2_NC. The results in Figure S9 show that it induced an absorbance change of less than 5% compared with data without CAT. Therefore, it is believed that the impact of catalase-like activity, if any, of nanoceria would be negligible in the MV experiment.

Other than the calatase-like activity of nanoceria, the possible reaction between H_2_O_2_ and CeO_2_ can also partly contribute to the enhanced hydroxyl radical scavenging ability of thermally treated ceria samples. Recent studies have suggested that ceria nanoparticles can be directly used to detect H_2_O_2_ and that their sensibility toward H_2_O_2_ is size-dependent.^49−51^ For ceria nanoparticles of <40 nm size, a partial reversible phase transformation can occur at the surface in the presence of highly concentrated H_2_O_2_ aqueous solution. The larger the particle size, the less reactivity between ceria and H_2_O_2_. In this study, Table 1 shows that the average particle sizes, for both CeO_2_ and 10% Pr-CeO_2_NC, increased from 22 and 27 nm to 31 and 37 nm, respectively. As it can be seen in Figure S2, the percentage of nanocubes bigger than 40 nm for CeO_2_NC and 10% Pr-CeO_2_NC is very small (around 1 and 9%). After oxidation at 600 °C, the percentage of nanocubes bigger than 40 nm of these two samples increases to 11 and 20%, respectively. This suggests that the size increase after oxidation of ceria nanocubes would not result in the dramatic change of reaction between H_2_O_2_ and ceria nanocubes. Thus, with combination of catalase results, the antioxidant activity on ceria nanocube samples and enhancement of antioxidant activity after oxidation at 600 °C cannot be only due to direct reaction between H_2_O_2_ and ceria nanocubes.

Finally, the phosphatase activity of the ceria nanocubes was measured by the paraoxon degradation experiment. Phosphatase, or phosphotriesterase (PTE), is one of the key enzymes in the cell signal transduction system. Bacteria uses it to break down organophosphorus compounds in residue of pesticides or chemical warfare agents.^28^Figure 6e illustrates the UV–vis spectra of paraoxon (0.05 M) with and without the presence of 5 mg mL^–1^ (∼29 mM) ceria nanocubes. The specific absorbance peak of degraded product *p*-nitrophenol at 401 nm shows that ceria NCs can catalyze the hydrolysis of paraoxon in NMM solution, in agreement with previously reported results for vacancy-enhanced ceria nanoparticles.^10^ It can be seen in Figure 6f that under the same conditions, the hydrolysis efficiency of four representative nanocubes are in the order 10% Pr-CeO_2_NC-O600 > 10% Pr-CeO_2_NC > CeO_2_NC-O600 > CeO_2_NC. In addition, the oxidized samples are better than the original samples, ∼4-fold for CeO_2_NC and 11% higher for 10% Pr-CeO_2_NC. The same tendency occurs when the absorbance was normalized by the surface area of the samples, as in Figure S8c. For 5% Pr-CeO_2_NC and 15% Pr-CeO_2_NC catalysts, the oxidized Pr-doped ceria nanocubes present higher hydrolysis efficiency than the original ones (Figure 6f).

It is noted that the hydrolysis efficiency above is not as high as previously reported by Mugesh et al., who managed to hydrolyze >80% paraoxon in similar conditions.^10^ This may be due to the different synthetic conditions for their vacancy-enhanced ceria nanoparticles. In order to figure out the possible role of Ce^3+^ in the hydrolysis reaction, as also suggested previously,^10,52^ 29 mM Ce^3+^ and Ce^4+^ were added to paraoxon in NMM solution, respectively. However, hydrolysis efficiencies of Ce^3+^ or Ce^4+^ are very low (Figure S10), even if at much higher Ce^3+^ concentration than their study.^10^ It suggests that isolated Ce^3+^ ions in solution present poor catalytic activity to degrade paraoxon, and the phosphatase activity of ceria nanocubes may be not solely or directly associated with the Ce^3+^ ions in the aqueous solution. The degradation of organophosphorus occurs on the surface of ceria nanocubes. The Ce^3+^ and Pr^3+^ species on the surface of these catalysts play an important role in this artificial enzymatic reaction. The mechanism will be discussed later.

## 4 Discussion

Taken together, the above-mentioned results suggest that a thermal treatment to the ceria nanocubes can improve the multienzyme-mimic activities, with and without Pr modification. Herein, the effect of Pr-doping will be discussed considering specifically how the oxidation treatment at 600 °C can significantly enhance the activity by altering the bulk structure regularity and inducing surface nanofaceting. The possible roles of oxygen vacancies, Ce^3+^ and Pr^3+^ defects, and band gap energy are considered and discussed. Possible mechanisms of ceria nanocubes performing different biomimetic functions are also proposed.

### 4.1 Mechanism of the Multienzyme-Mimic Activities

It is well-known that the redox, in the form of oxidizing and reducing, properties of ceria is closely related to oxygen vacancies (VO^••^) and reactive oxygen species (ROS). Numerous studies^1,2,4^ have shown that oxygen species and oxygen vacancies are crucial in ceria-based nanozyme performance, such as the role of superoxide O_2_^•–^ for oxidase-like^53^ and the hydroxyl radical (OH^•^) scavenging activities.^18^ In the face centered cubic fluorite CeO_2_ structure, Ce^4+^ cations are coordinated by eight oxygen atoms, whereas oxygen atoms are tetrahedrally linked with cerium atoms. With the formation of Ce^3+^ or incorporation of Pr^3+^, Ce^4+^ cations in the lattice are replaced by the trivalent Ce^3+^ or Pr^3+^ cations. The oxygen coordination number is decreased to seven to maintain the charge neutrality and stoichiometry of bulk ceria. The removed oxygen generates a VO^••^ in the crystalline structure of ceria. The concentration of VO^••^ directly influences the oxygen diffusion and migration to the surface, which consequently affect the oxygen availability, oxygen regeneration capacity, and thus redox kinetics on the surface of the catalysts.

**Figure 7:**
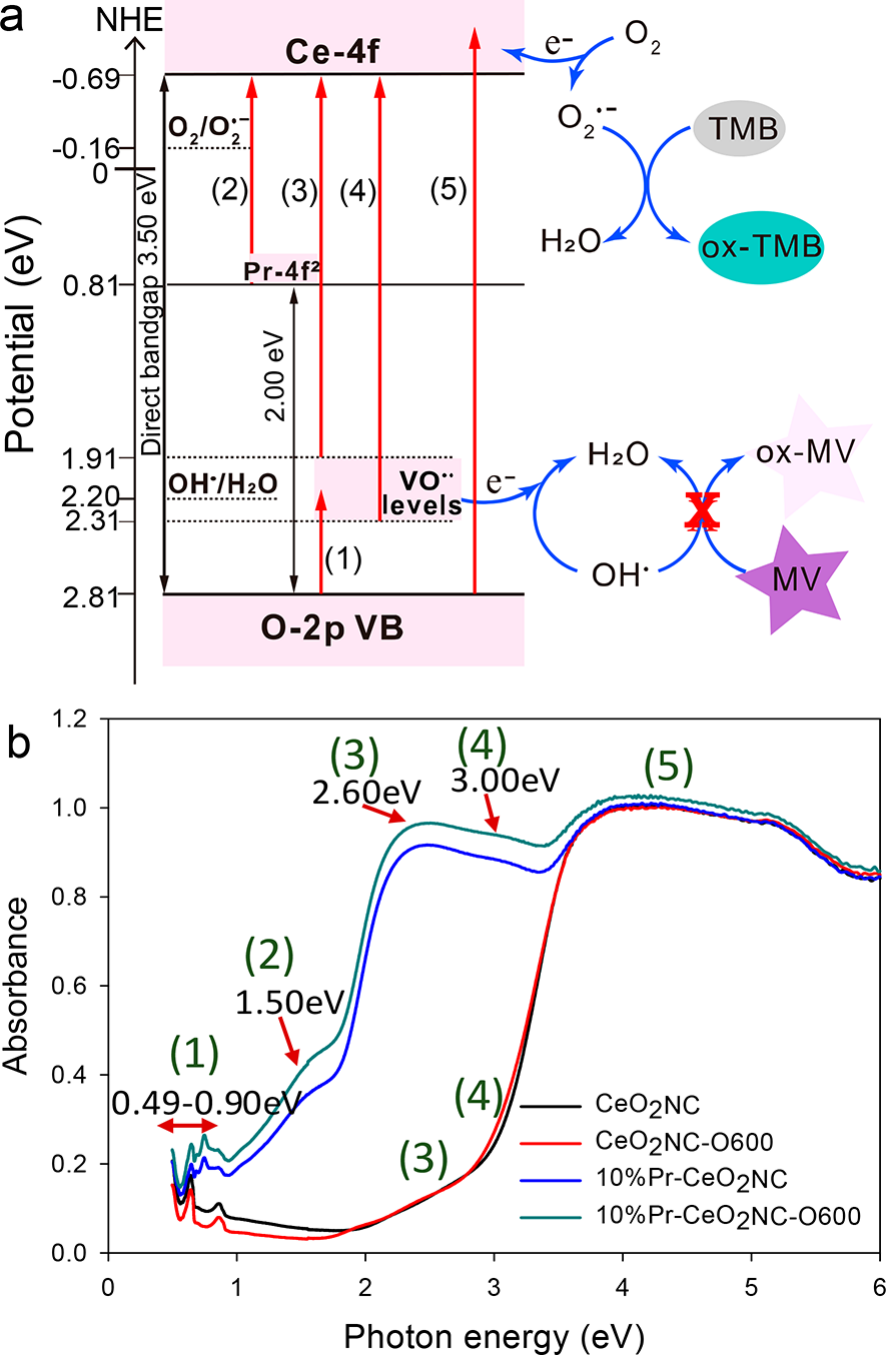
(a) Energy level diagram and excitation processes of pure and Pr-doped CeO_2_ nanocubes; (b) absorbance spectra of CeO_2_NC and 10% Pr-CeO_2_NC samples with and without 600 °C oxidation treatment.

The oxidase-like activity, that is, the oxidizing capability, of ceria has been reported previously.^21,53^ During the reaction, it was found that the main intermediate is superoxide O_2_^•–^.^53^ The dissolved O_2_ molecules can be adsorbed on the surface of ceria nanocubes, and a 4f electron feedbacks to the π2p* orbital of O_2_ to form a superoxide O_2_^•–^. O_2_^•–^ continues to oxidize TMB into blue-colored ox-TMB. The simplified pathway can be explained in the form of an electronic band diagram in Figure 7a. Based on eqs 2 and 3 and using *E*_g_ = 3.5 eV (energy of direct band gap of CeO_2_ NC in Table 2), the valence band energy *E*_VB_ of CeO_2_NC can be calculated to be 2.81 eV, in which χ = 5.56 eV is the absolute electronegativity (AEN) of CeO_2_ and *E*^C^ = 4.5 eV is the scaling factor relating the normal hydrogen electrode scale (NHE) to the absolute vacuum scale.^21^
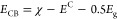
2
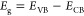
3

Generally, the energy levels of adsorbed and potentially formed ROS can be approximated by their standard redox potential *E*_0_. The feasibility of electron transfers between ceria and adsorbed ROS can be determined by the relative energy position of *E*_VB,_*E*_CB_ and *E*_0_. In aqueous media, the redox potential *E*_0_ for the dissolved O_2_/O_2_^•–^ couple is −0.16 eV, while it is 2.2 eV for the OH^•^/H_2_O couple.^54^

Because *E*_CB_ of ceria (−0.69 eV) is more negative than the potential of O_2_/O_2_^•–^ (−0.16 eV), the conduction band electrons are good reductants, so that O_2_ can be reduced to generate O_2_^•–^, which consequently oxidizes TMB. Furthermore, Pr-doping in CeO_2_ can create excited *f*-energy states in the midband as elucidated in the optical absorption spectra of Figure 7a. The distance between the top of the O 2p VB and Pr 4f^2^ band according to the calculated direct band gap energy for Pr_6_O_11_ is 2 eV (Figure S7), resulting in the narrowing of band gap. The energy state of the Pr 4f^2^ band can trap electrons from O_2_ easily, which is one of the reasons why 10% Pr-CeO_2_NC is more active as oxidase-mimic than pure ceria nanocubes as shown in Figure 6a,b.

Meanwhile, oxygen vacancies in ceria nanocubes may explain the hydroxyl radical scavenging activity. According to Choudhury et al.,^32^ oxygen vacancies are located between the band gaps of Ce 4f and O 2p, in the band gap of CeO_2_, again this intermediate defect energy level results in the narrowing the band gaps. The level of oxygen vacancies can be estimated from the photon energy profile based on UV–vis absorbance data in Figure 3. As shown in Figure 7b, each absorption band can be associated with an electron transition between two energy states in Figure 7a. For example, the absorption bands centered at 2.6 and 3 eV, i.e., 1.91 and 2.31 eV vs NHE, could be assigned to the charge transfer transition from oxygen vacancies to Ce 4f CB. The intensity of these peaks is higher for Pr-doped sample due to the Pr presence which promotes the production of oxygen vacancies. The standard redox potential *E*_0_ OH^•^/H_2_O couple is 2.2 eV,^54^ and the OH^•^ radicals generated from the Fenton reaction can be reduced to H_2_O by accepting electrons from the VO^••^ band. It has been previously reported that the redox state of ceria nanoparticles would not change with pH;^55^ therefore, redox potentials are quoted at neutral pH, although the oxidation and antioxidant experiments took place in more acidic conditions.

The reaction chemistry for phosphatase-mimic activity of ceria was reported to do with the mixed Ce^3+^/Ce^4+^ oxidation states. The surface Ce^4+^ ions allow the binding of paraoxon because of the polar nature of phosphoryl oxygen, meanwhile Ce^3+^ would facilitate the selective adsorption of H_2_O. Consequently, the steric coordination enables an efficient nucleophilic attack of water at the phosphorus center of paraoxon and hence the P–O bond cleavage. It implies that the concentration of Ce^3+^–O–Ce^4+^ defect sites as well as oxygen vacancies that are coordinately unsaturated are important in the hydrolysis of toxic paraoxon. Although both Pr and Ce are rare earth metals known for forming numerous nonstoichiometric oxides, their stable phases are different, CeO_2_ for Ce and Pr_6_O_11_ for Pr. It means that varying ratios of Ce^3+^/Ce^4+^ or Pr^3+^/Pr^4+^ together with the corresponding oxygen vacancies to balance the charges. Therefore, it is expected that doping CeO_2_ with Pr should increase the total trivalent ions concentration as well as the VO^••^ concentration, which was confirmed by the Raman peak at 572 cm^–1^ (Figure 4). In addition, XPS results indicate that all Pr species on the surface of 10% Pr-CeO_2_NC are Pr^3+^, while only a small amount of Pr^4+^ can be found for the oxidized 10% Pr-CeO_2_NC-O600 sample. It also should be mentioned that the incorporation of Pr to ceria nanocubes enhances the redox property of pure ceria nanocubes, as shown in the TPR results (Figure 5), which also is favorable to redox Ce^3+^/Ce^4+^ and Pr^3+^/Pr^4+^ couple cycles and then improves the phosphatase-mimic activity of ceria nanocubes. All these effects contribute to the enhanced hydrolysis efficiency of phosphatase-like activity of 10% Pr-CeO_2_NC.

### 4.2 Influence of Oxidation Treatment at 600 °C

**Figure 8:**
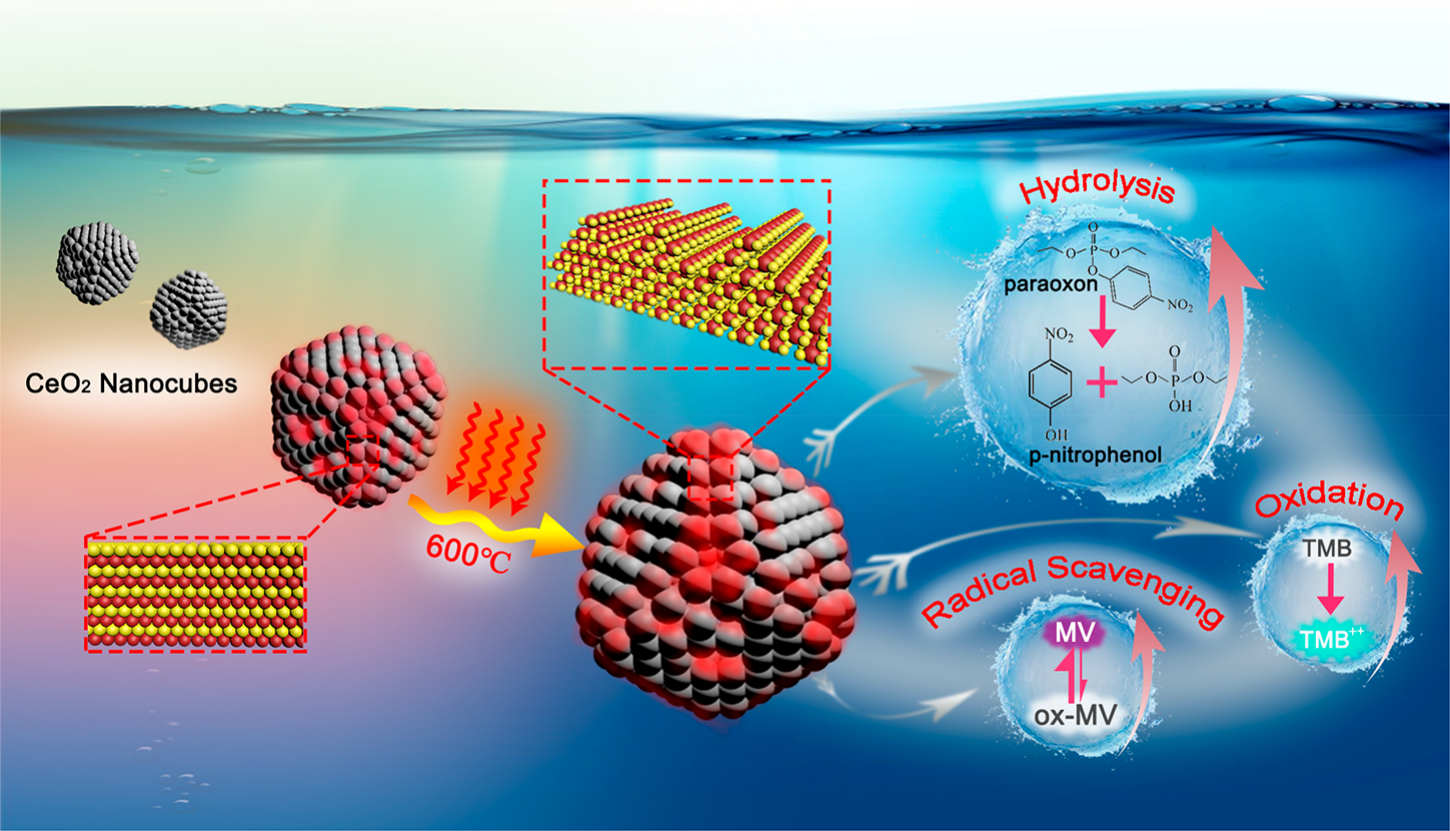
Schematic illustration of how the {110} and {111} nanofacets are generated on {100}-dominant CeO_2_NC surfaces via an oxidation treatment at 600 °C, which consequently enhance their biomimetic enzyme activities.

In general, the biomimetic enzyme activity results in Figure 6 show that the oxidized ceria nanocube samples exhibit higher capability in imitating oxidase, hydroxyl radical scavenger, and phosphatase than the untreated NCs. After discussion of the possible mechanism of ceria acting as three types of artificial enzymes, the influence of thermal oxidation (5% O_2_/He at 600 °C for 1 h) on ceria’s nanocube structure and biomimetic enzyme activities and their correlation are considered and shown in Figure 8.

As deduced from TEM, XPS, and XRD results, most of the bulk properties of the samples, such as lattice parameter, Pr concentration, and Ce^3+^/Ce^4+^ ratio, are almost the same after thermal treatment (Table 1). There is, indeed, a crystal size increase and, consequently, a BET specific surface area drop, which is probably due to the heat-induced aggregation. The most remarkable structure change is the facet restructuring on the crystal surface, including the facet percentage change and the development of sawtoothlike nanofacets (Figure 1 and Figure S3). After oxidation at 600 °C, the percentage of {110} increases dramatically, 50% for CeO_2_NC and 118% for 10% Pr-CeO_2_NC. At the same time, the proportion of {100} reduces together with negligible change of {111} (inset table in Figure 1). This is reasonable because the {100} surface is a polar surface and not stable upon heating.^56,57^ Oxidation at 600 °C leads to relaxation from {100} facets to low energy {110} and {111} facets, which often appear with defects.

The restructuring of the surface into nanofacets can cause a series of effects. The first is the change of crystallographic termination groups. For instance, it was known that the density of oxygen atom varies on different ceria facets, following the order {111} (15.8/nm^2^) > {100} (13.7/nm^2^) > {110} (9.7/nm^2^).^58^ So more clustered defect sites are expected on {110} than on {100}. This is confirmed by previous UV Raman results that nanorods with exposed {110} and {100} surfaces have the most defect sites, followed by nanocubes with {100} and nano-octahedra with {111}.^43^ The difference in defect sites results in changes in the stability and reactivity of O_2_ or ROS adsorbed on the nanocrystal surface. Density functional theory calculation showed that the binding energy of superoxide O_2_^•–^ on ceria {110} surface is much larger than those of other surfaces.^59,60^ In this study, both CeO_2_NC-O600 and 10% Pr-CeO_2_NC-O600 have increased proportion of {110} facets. This may account for their increased oxidase-like behavior in the TMB oxidation experiment.

Another change of surface termination on different facets is related to Ce atoms. Ce cations are more exposed on the {110} surface than on the O-terminated facets of the {100} surface. As discussed in section 4.1, the phosphatase-like activity of ceria surface origins from the Ce^3+^-bound water initiating the nucleophilic attack at the phosphorus center of Ce^4+^-bound paraoxon. Therefore, the increase of exposed Ce would facilitate the reaction kinetics, that is, a boost of the phosphatase-like activity.

The final effect is about the increased level of oxygen vacancies in the oxidized ceria samples, based on UV–visible (Figure 3) and Raman spectra (Figure 4). The higher presence of oxygen vacancies provides more freedom for the movement of lattice oxygen and hence increases oxygen mobility in ceria, i.e., its capability to buffer oxygen.^19,61^ Theoretical calculation has predicted that the oxygen vacancy formation energy on ceria follows the order {110} < {100} < {111}.^62^ In other words, VO^••^ is the easiest to form on {110}. Hence it is likely that the oxidized ceria nanocubes with higher ratio of {110} has more oxygen vacancies to intermediate the redox reactions.

The nature of the defect sites is critical for understanding the catalysis and the oxidation-induced activity enhancement. The defects in ceria have been discussed in many previous studies. Some attribute them to the presence of Ce^3+^ while others relate them with oxygen vacancies. In this study, XPS spectra of our four ceria nanocubes (Figure S6) show that Ce 3d profiles are very similar. Further calculation indicates that even if there is Ce^3+^ present on the surface, the amount of Ce^3+^ is small (<9%) and similar on the four different ceria nanocubes. In other words, it seems that Ce^3+^/Ce^4+^ ratio has insignificant correlation to the observed trend in our study. This is in contrast to some previous studies such as Celardo et al.^22^ showing that Ce^3+^/Ce^4+^ ratio is crucial in Sm-doped nanoceria as antioxidant, but agrees with other research results where nanoceria with little or similar Ce^3+^ percentage but different morphology could have dramatic different catalytic activities.^18,21,63,64^ However, it should be highlighted that on the Pr-doped ceria nanocube samples, 100% Pr is in the oxidation state of Pr^3+^ as shown in XPS results, that is 10% to the total Ce and Pr amounts. Similar to Ce, the redox Pr^3+^/Pr^4+^ cycle can also be considered as an oxygen buffer. Hence, the total amount of Ce^3+^ and Pr^3+^ on 10% Pr-CeO_2_NC is 12%, which is slightly higher than in the pure CeO_2_NC sample. For the oxidized 10% Pr-CeO_2_NC-O600 sample, only a small amount of Pr^4+^ was observed according to XPS results. This suggests that there is slightly less than 10% Pr^3+^ in this sample. In any case, in this study the vacancy-interstitial (Frenkel-type) oxygen defects cannot be excluded to explain our results.^43,65,66^ The intrinsic Frenkel-type defect is believed to form when some oxygen in ceria migrates from the tetrahedral site to the octahedral site, leaving vacancies in the tetrahedral sites. It is proposed that the interstitial oxygen ions are the “active” species that provide necessary oxygen mobility, which is crucial in the function of ceria as a catalyst. Mamontov et al.^65^ showed that for pure CeO_2_ both oxygen vacancies and the interstitial oxygen ion concentration, as well as the total oxygen defect concentration, increase with temperature until they reach the maximum at ∼600 °C. Another separate study on the dephosphorylation reaction of several nucleotides like adenosine triphosphate by ceria particles showed that the most effective ceria are those calcined at 600 °C.^67^ These results are consistent with the influence of oxidation of ceria nanocubes with and without Pr doping observed in this study.

In summary, the oxidation treatment leads to the formation of the Frenkel-type oxygen defects, surface restructuring, and changes of surface facets. Therefore, it causes the reactivity of adsorbed oxygen species to increase, an increase of the oxygen vacancy concentration, and more exposed Ce^3+^, which eventually result in the activity enhancement of CeO_2_ nanocubes as oxidase, hydroxyl radical scavenger, and phosphatase, respectively. It is worth noting that the majority of previous work on ceria as an artificial enzyme uses polycrystalline nanoceria. It is known that different crystal planes of ceria exhibit different surface structures, interactions, and catalytic efficiencies. Furthermore, the knowledge based on polycrystalline ceria is merely a collective average from different facets, which makes it difficult to correlate to the catalytic behavior. This study on restructured nanofacets helps us to gain fundamental understanding of ceria’s biomimetic function, which could potentially provide support for designing a better ceria-based nanozyme.

## 5 Conclusions

An oxidation treatment was established for {100}-dominant pure and Pr-doped ceria NCs with well-defined structures and surfaces to achieve a significant increase in their {110} and {111} nanofacets. More importantly, without changing most of the bulk properties, and despite the drop in BET specific surface area, such treatment results in a remarkable boost of their artificial enzyme activities imitating oxidase, hydroxyl radical scavenger, and phosphatase. This might be due to the thermally generated {110} nanofaceting and increased level of Frenkel-type oxygen defects, which facilitate the oxygen mobility and the formation of oxygen vacancies on the surface. This is very interesting because the seemly contradictory (oxidizing and reducing) behaviors can both be enhanced by an oxidation treatment. It offers a new approach, to the best of our knowledge, the first time reported, to improve the intrinsic enzymatic activity of nanoceria. Considering the wide application of ceria as an artificial enzyme, this simple process may provide great support for designing a high efficiency nanozyme without the change of crystal composition. It may even be extended to other metal-oxide based nanozymes, which is worthy of further investigation.

## Supporting Information Available

TEM or STEM-HAADF images, Ce and Pr element maps and particle size distribution of pure and Pr-doped ceria nanocubes, XRD patterns, XPS spectra of Ce 3d and Pr 3d, band gap energy determination of the samples by the Tauc method, artificial enzyme activities of oxidase, hydroxyl radical scavenger and phosphatase normalized by the BET specific surface area, role of catalase in hydroxyl radical removal, and NCs’ performance comparison with Ce ions (PDF)
