# Supplementary material for: Enhanced Artificial Enzyme Activities on the Reconstructed Sawtoothlike Nanofacets of Pure and Pr-Doped Ceria Nanocubes
Source: ACS Appl Mater Interfaces. 2021 Aug 9;13(32):38061–73. doi: 10.1021/acsami.1c09992 (PMC8674880; doi:10.1021/acsami.1c09992)
Supplement: Supplementary file 1 — am1c09992_si_001.pdf [file am1c09992_si_001.pdf]

Supporting Information

Enhanced artificial enzyme activities on the reconstructed sawtooth-like nanofacets of pure and Pr-doped ceria nanocubes

Lei Jiang[b,*], Miguel Tinoco[a], Susana Fernández-García[a], Yujiao Sun[b], Mariia Traviankina[b], Pengli Nan[b], Qi Xue[b], Huiyan Pan[a, d], Almudena Aguinaco[c, e], Juan M. González-Leal[c, e], Ginesa Blanco[a, e], Eduardo Blanco[c, e], Ana B. Hungría[a, e], Jose J. Calvino[a, e], Xiaowei Chen[a, e,*]

[a] Departamento de Ciencia de los Materiales, Ingeniería Metalúrgica y Química Inorgánica, Facultad de Ciencias, Universidad de Cádiz, Campus Río San Pedro, Puerto Real (Cádiz), E-11510, Spain

[b] Heavy Oil State Laboratory and Center for Bioengineering and Biotechnology, College of Chemical Engineering, China University of Petroleum (East China), Qingdao, 266580, China

[c] Departamento de Física de la Materia Condensada, Facultad de Ciencias, Universidad de Cádiz, Campus Río San Pedro, Puerto Real (Cádiz), E-11510, Spain

[d] Henan Key Laboratory of Industrial Microbial Resources and Fermentation Technology, College of Biological and Chemical Engineering, Nanyang Institute of Science and Technology, Nanyang, 473004, China

[e] Instituto Universitario de Investigación en Microscopía Electrónica y Materiales (IMEYMAT), Universidad de Cádiz, Campus Río San Pedro, Puerto Real (Cádiz), E-11510, Spain

* Emails: xiaowei.chen@uca.es; leijiang@upc.edu.cn

Table S1. Physicochemical properties of the 5%Pr-CeO_2NC and 15%Pr-CeO_2 samples

Sample	BET surface areas (m^2 g^{-1})	Composition by ICP (mol%)		Composition by XPS (mol%)		Average particle size (nm) [a]	Scherrer τ (nm) [b]	Lattice parameter (Å) [b]
		Ce	Pr	Ce	Pr			
5%Pr-CeO_2NC	26	95.6	4.4	88.1	11.9	31	24	5.4
15%Pr-CeO_2NC	23	85.6	14.4	74.9	25.1	27	32	5.4

[a] Calculated from the size distribution, including around 100 nanoparticles, obtained by TEM.

[b] Calculated by Scherrer equation using XRD data.

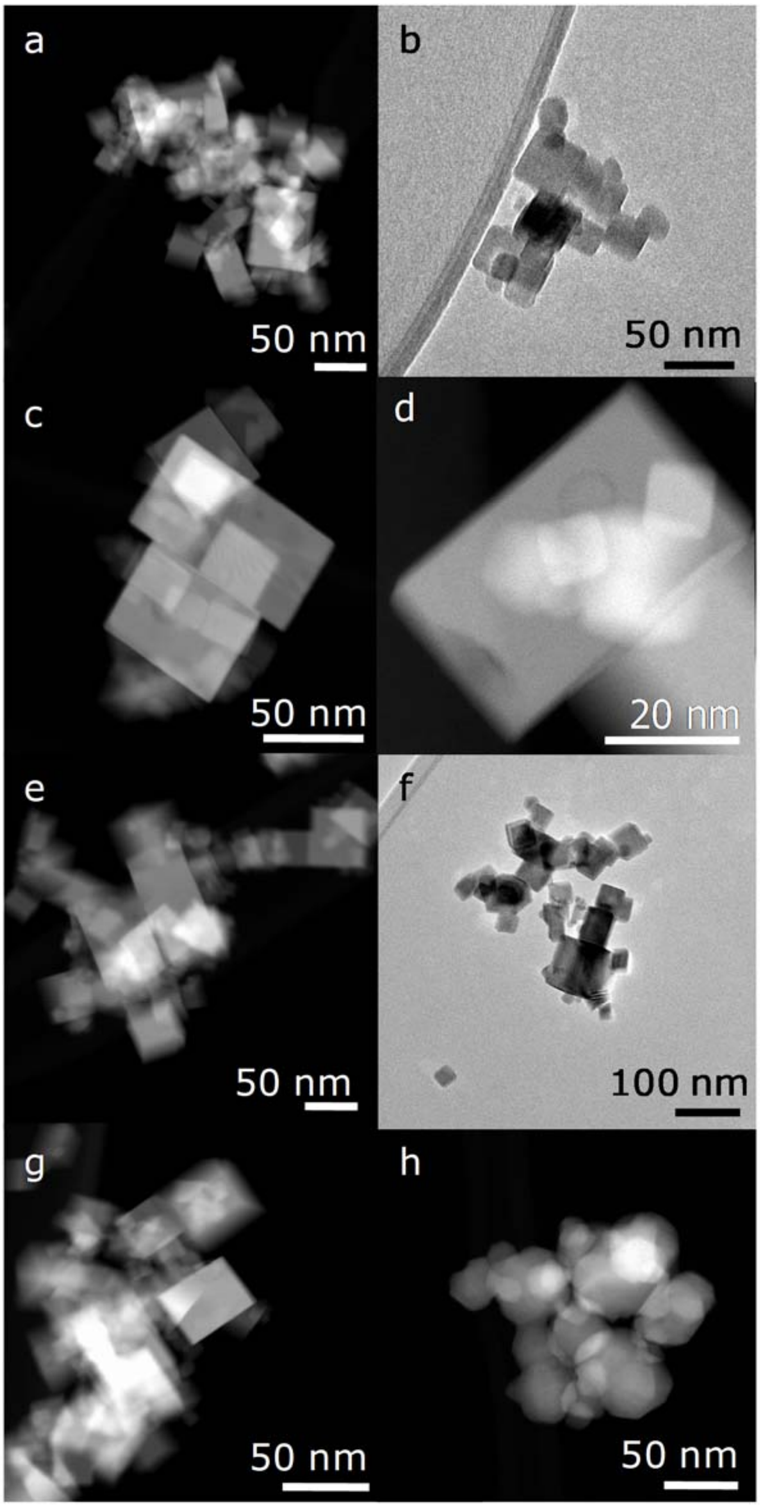

Figure S1. Low magnification (S)TEM images of pure and Pr-doped CeO_2 NCs. (a) STEM-HAADF image of CeO_2NC, (b) TEM image of CeO_2NC-O600, (c-e) STEM-HAADF images of 5%Pr-CeO_2NC, 5%Pr-CeO_2NC-O600, and 10%Pr-CeO_2NC samples, respectively, (f) TEM image of 10%Pr-CeO_2NC-O600, (g-h) STEM-HAADF images of 15%Pr-CeO_2NC and 15%Pr-CeO_2NC-O600 samples, respectively.

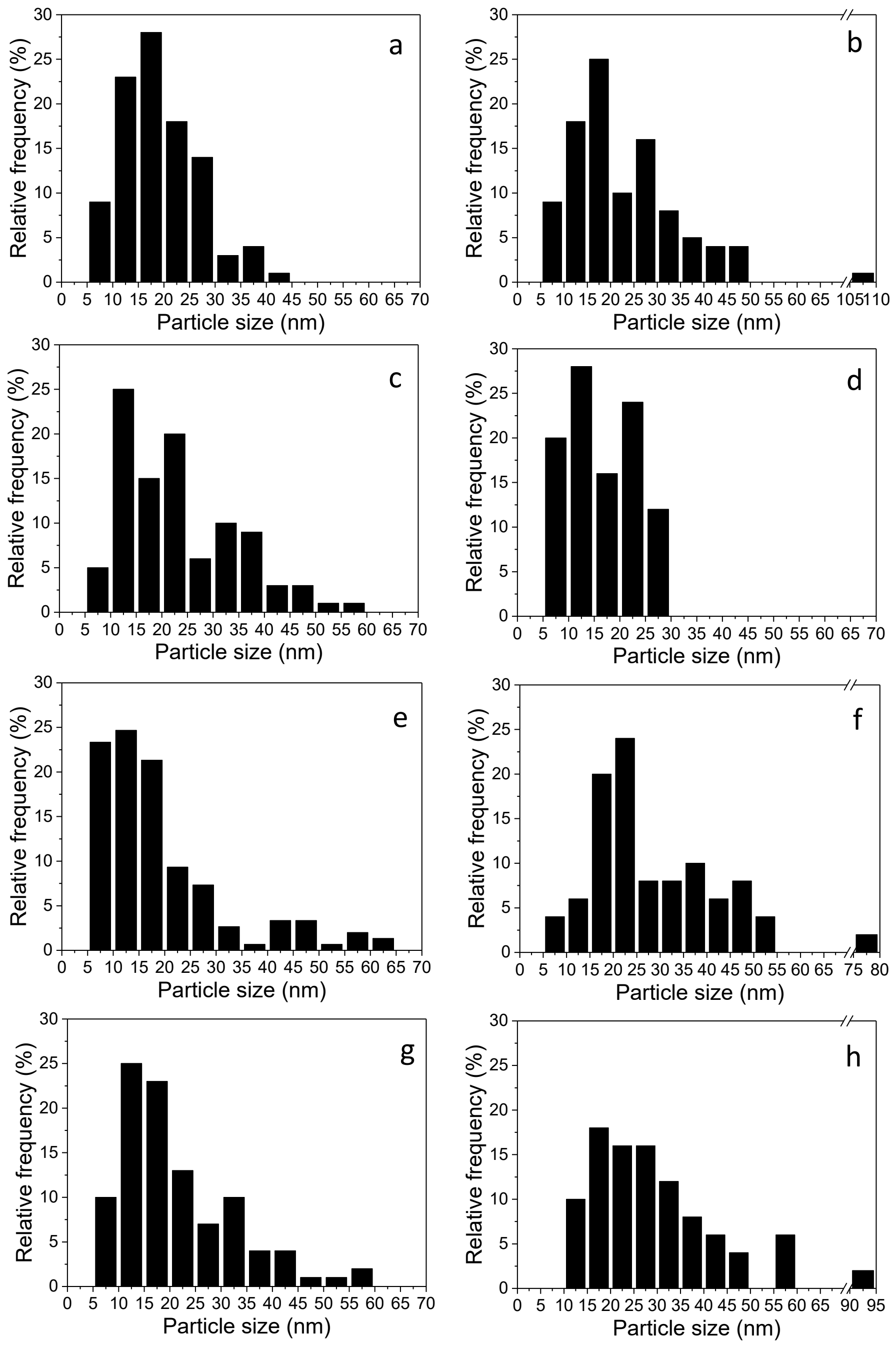

Figure S2. Particle size distributions obtained by measuring 25-150 nanocubes in samples (a) CeO_2NC, (b) CeO_2NC-O600, (c) 5%Pr-CeO_2NC, (d) 5%Pr-CeO_2NC-O600, (e) 10%Pr-CeO_2NC, (f) 10%Pr-CeO_2NC-O600, (g) 15%Pr-CeO_2NC, and (h) 15%Pr-CeO_2NC-O600.

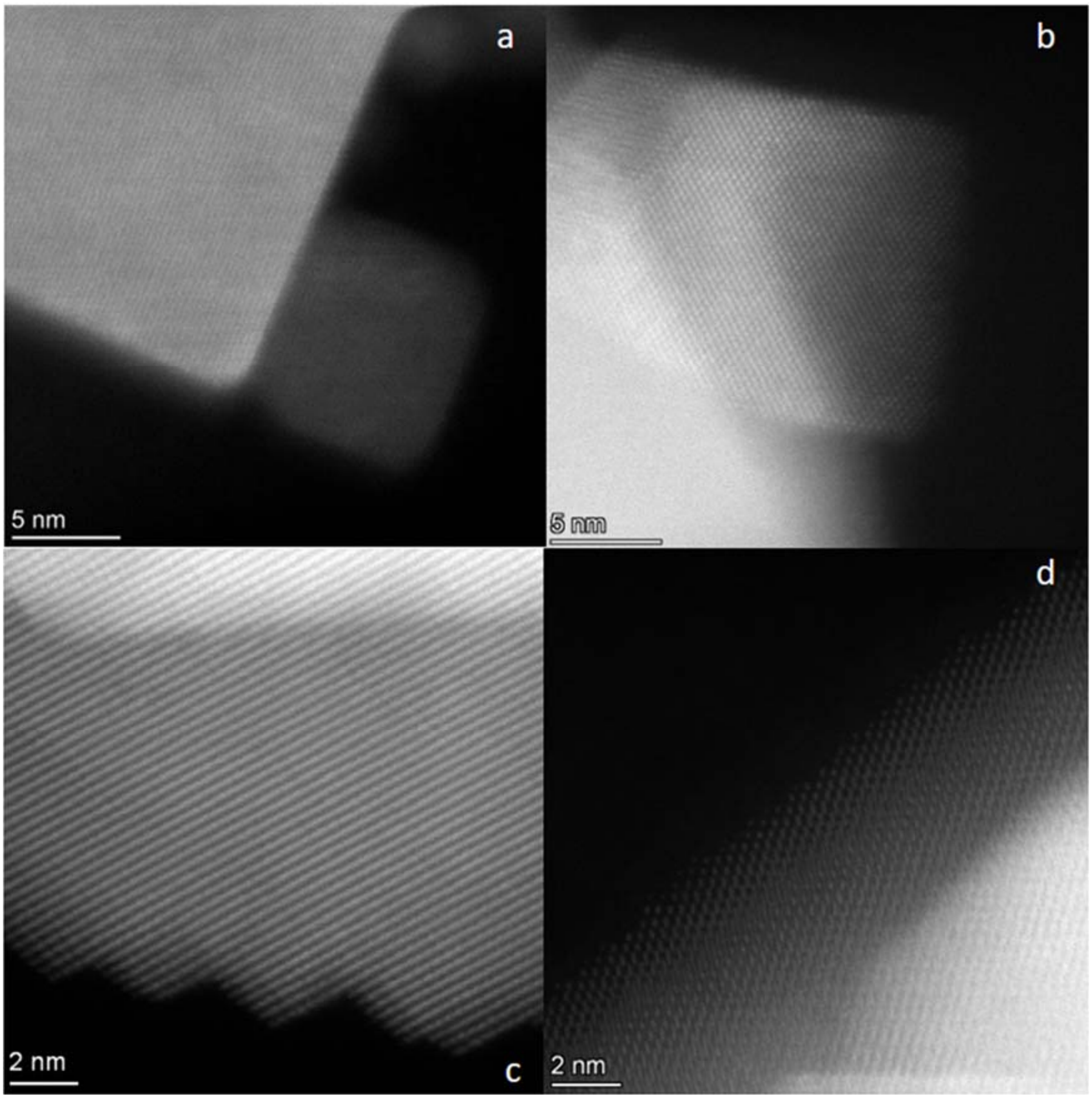

Figure S3. High resolution STEM-HAADF images of (a) 5%Pr-CeO_2NC, (b) 15%Pr-CeO_2NC, (c) 5%Pr-CeO_2NC-O600 and (d) 15%Pr-CeO_2NC-O600 samples.

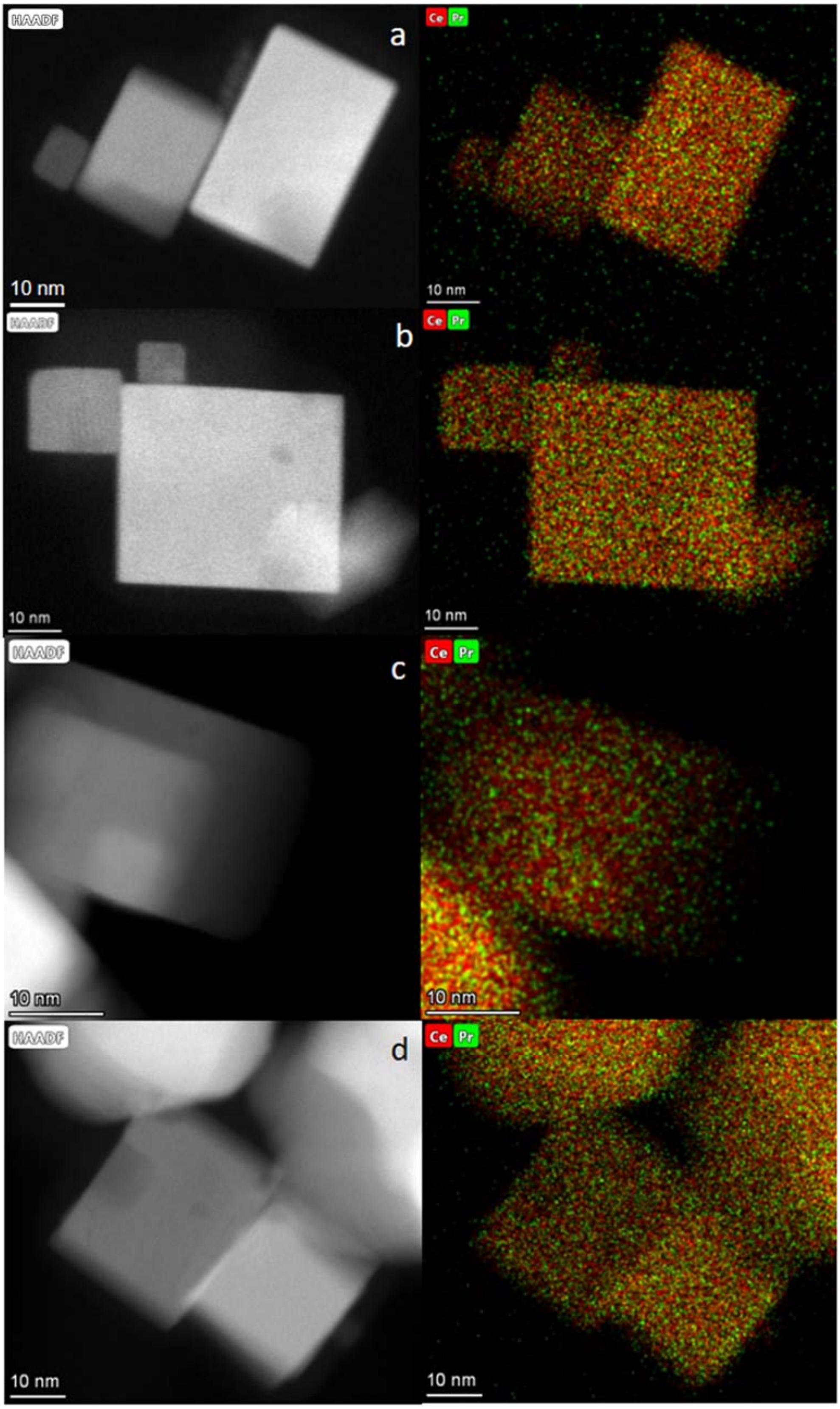

Figure S4. STEM-HAADF images and XEDS element maps of Ce (red) and Pr (green) of (a) 5%Pr-CeO_2NC, (b) 15%Pr-CeO_2NC, (c) 5%Pr-CeO_2NC-O600 and (d) 15%Pr-CeO_2NC-O600 samples.

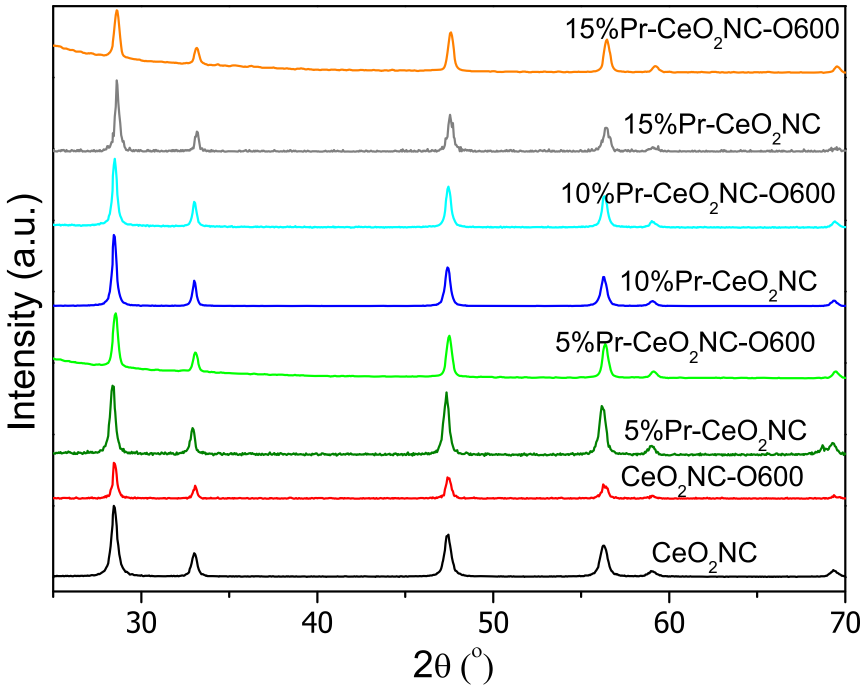

Figure S5. XRD patterns of the ceria nanocube samples.

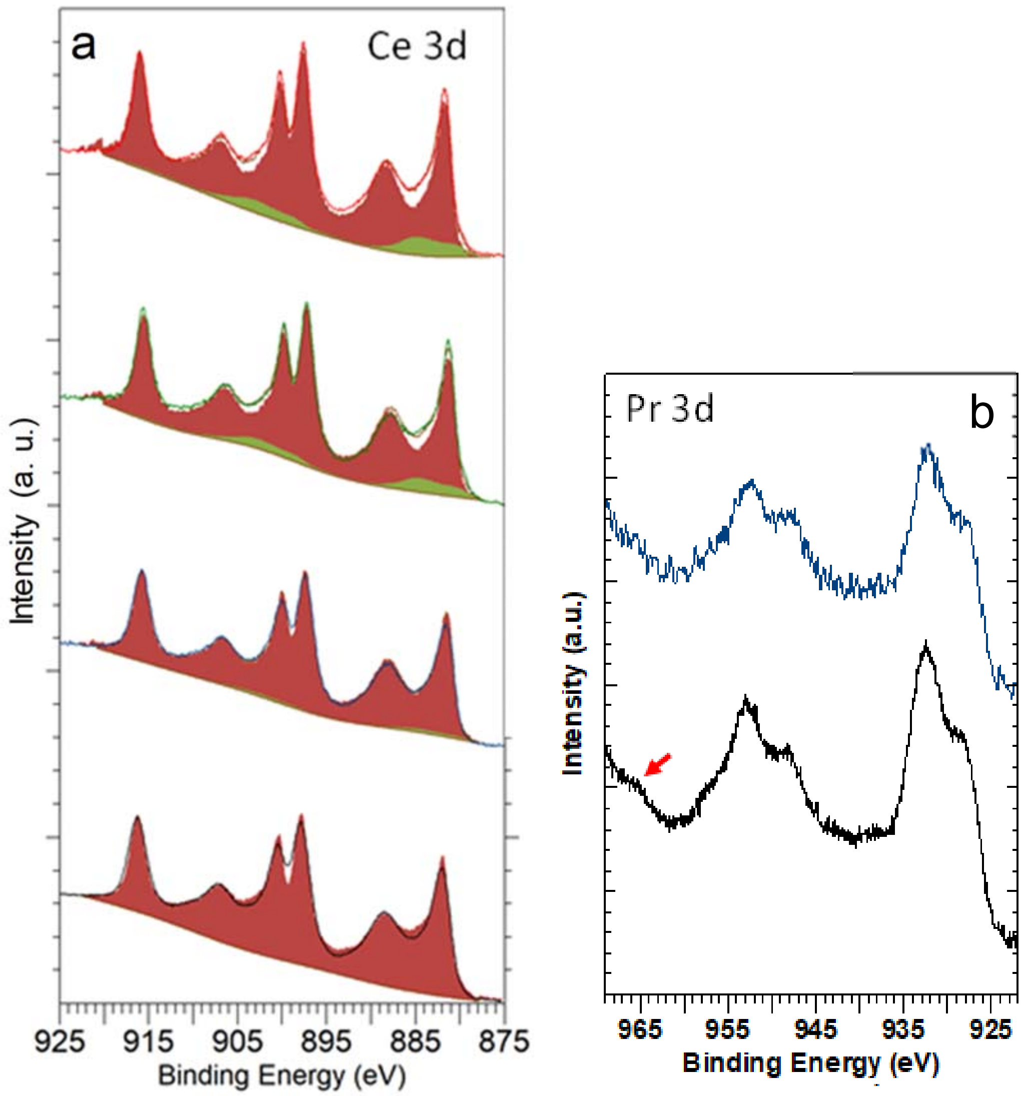

Figure S6. XPS spectra of (a) Ce *3d* and (b) Pr *3d* of CeO_2NC (red), CeO_2NC-O600 (green), 10%Pr-CeO_2NC (blue) and 10%Pr-CeO_2NC-O600 (black) catalysts, with contributions to Ce *3d* of Ce^{4+} (brown) and Ce^{3+} (green) oxidation states. Red arrow points the characteristic Pr^{4+} peak.

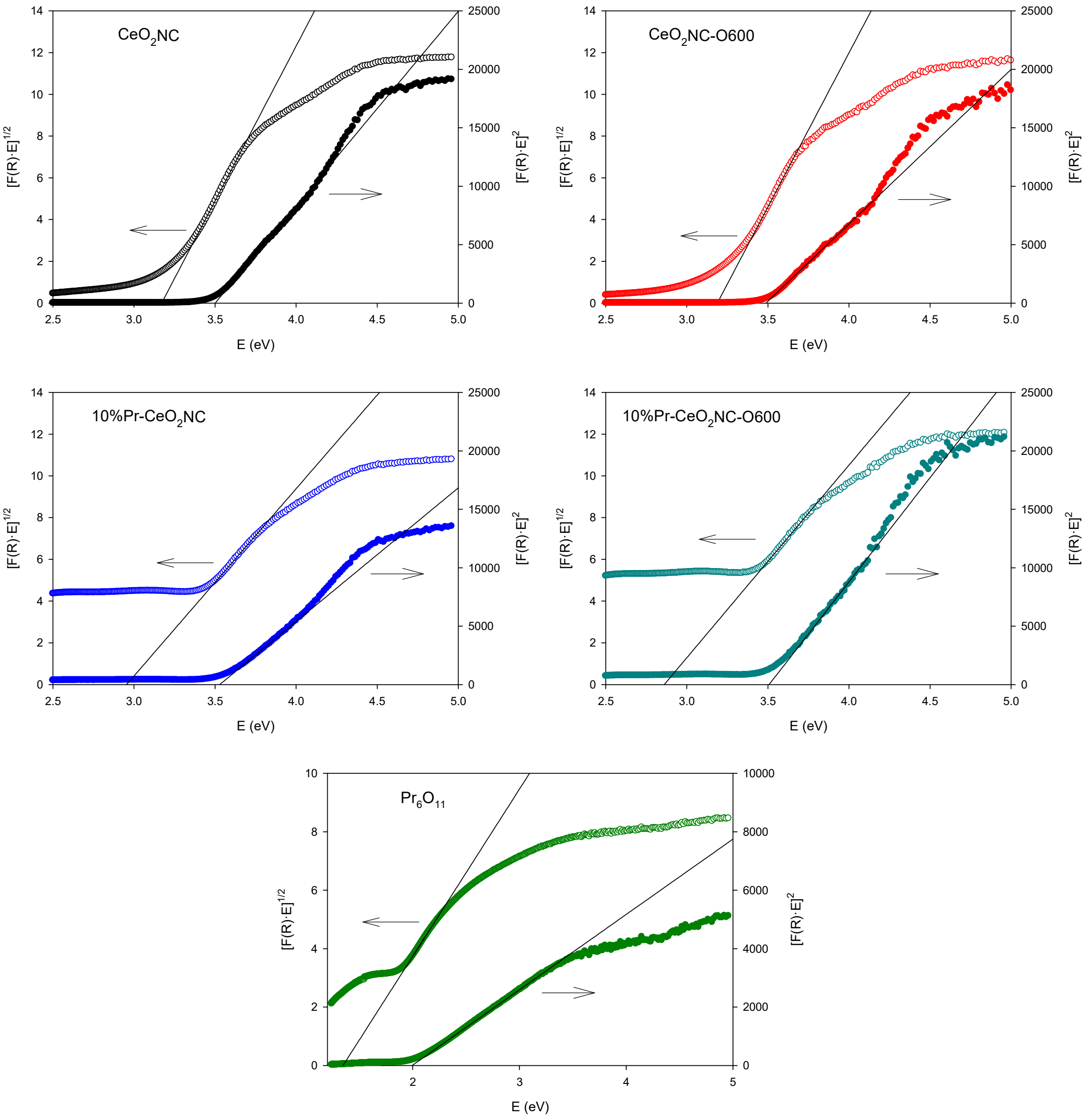

Figure S7. Band gap energy determination of the samples of CeO_2NC, CeO_2NC-O600, 10%Pr-CeO_2NC, 10%Pr-CeO_2NC-O600 and Pr_6O_{11} by Tauc method.

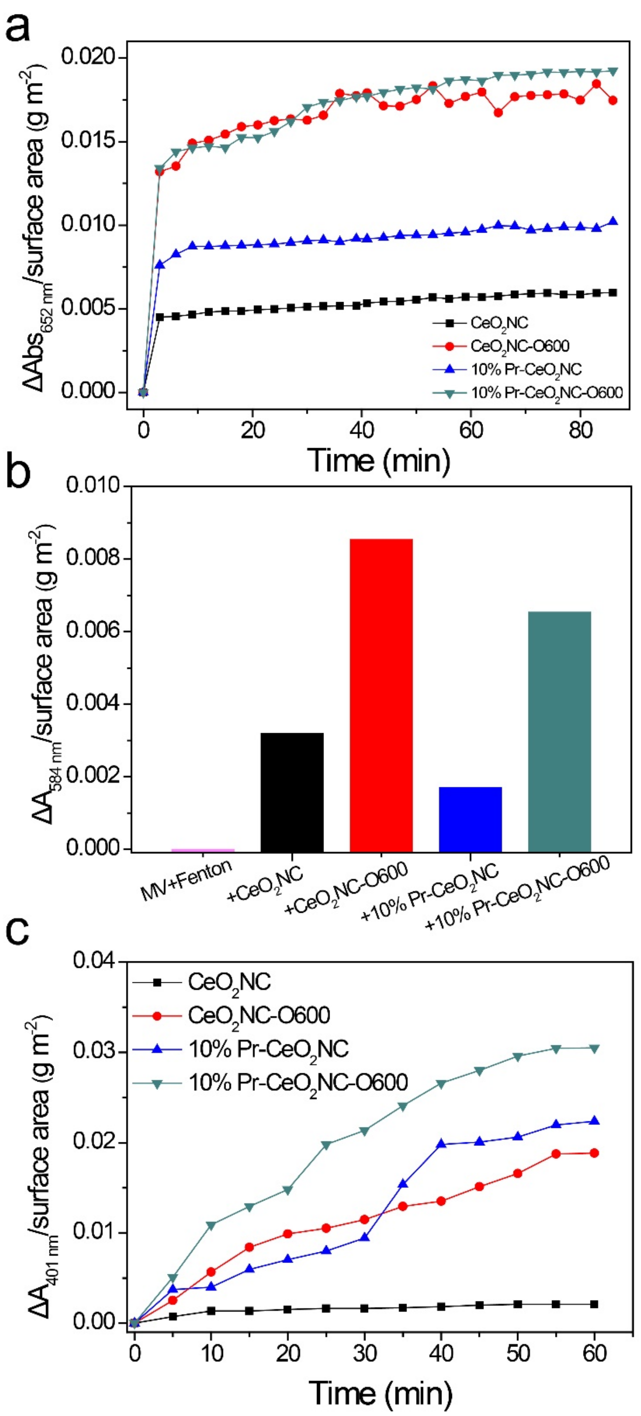

Figure S8. Comparison of CeO_2NC, CeO_2NC-O600, 10%Pr-CeO_2NC and 10%Pr-CeO_2NC-O600 samples for mimicking (a) oxidase, (b) hydroxyl radical scavenger and (c) phosphatase activities. The "Y" axis is the change of absorbance @652, 584 or 401 nm, respectively, normalized by the BET specific surface area of each sample.

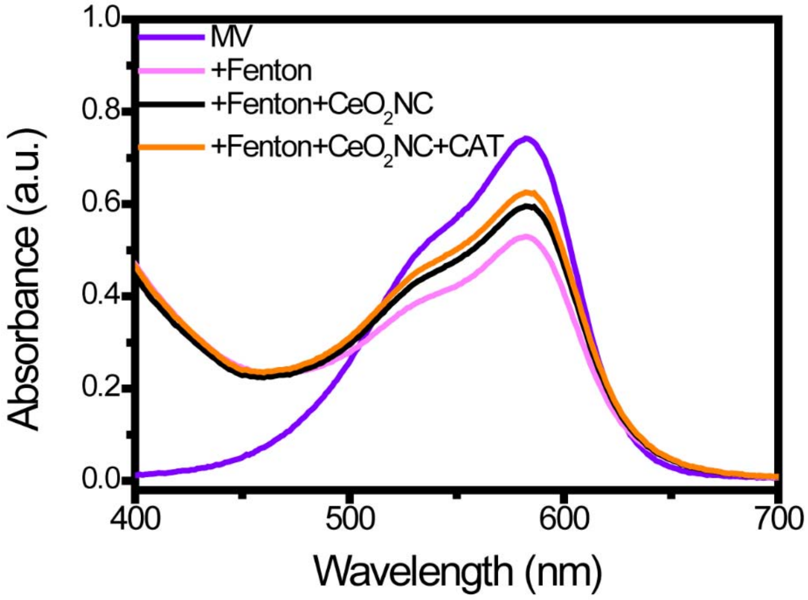

Figure S9. The UV-vis spectrum comparison of MV (2.4×10^{-5} M) after mixing with Fenton reagent ($FeSO_4$ and H_2O_2), with CeO_2NC (1.7 μg mL^{-1}) and the additional catalase (CAT, 21.1 μg mL^{-1} or 4.2 unit).

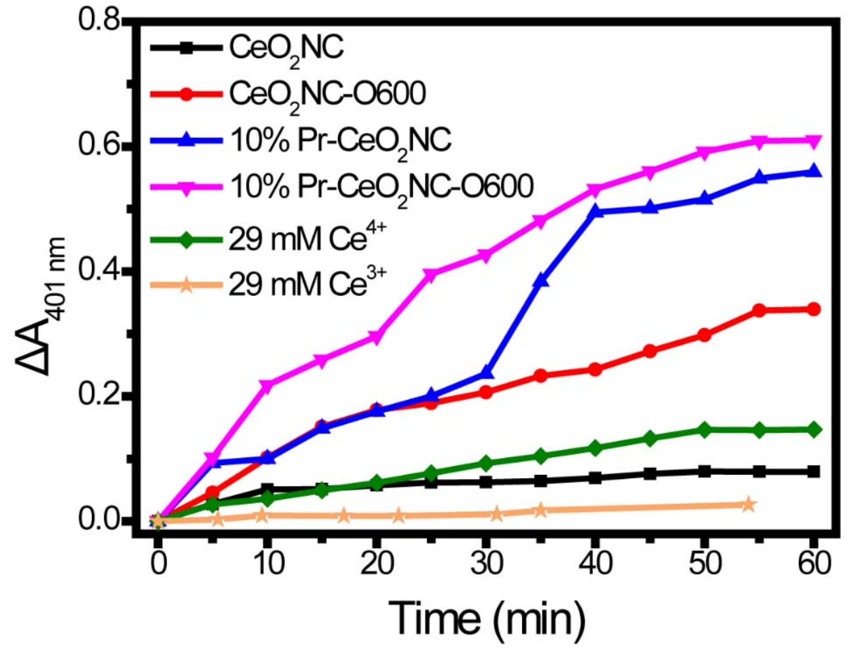

Figure S10. The UV-vis absorbance at 401 nm of the four ceria samples, as well as 29 mM $CeCl_3$ (Ce^{3+}) and $Ce(SO_4)_2$ (Ce^{4+}) solutions, in hydrolytically degrading 0.05 M paraoxon with time.
